# B cell-intrinsic Ssu72 phosphatase is a key regulator of allergic asthma

**DOI:** 10.1038/s12276-026-01859-0

**Published:** 2026-10-05

**Authors:** Seo-Young Koo, Naiyu Wang, Min-Hee Kim, Sujin Lee, Tae Jin Kim, Chang-Woo Lee

**Affiliations:** 1 Department of Molecular Cell Biology, Samsung Medical Center, Sungkyunkwan University School of Medicine, Suwon, Republic of Korea; 2 Department of Immunology, Sungkyunkwan University School of Medicine, Suwon, Republic of Korea; 3 Personalized Cancer Immunotherapy Research Center, Sungkyunkwan University, Suwon, Republic of Korea

**Keywords:** Signal transduction, Immunological disorders

## Abstract

B cell-derived immunoglobulin E (IgE) plays a crucial role in the development of allergic asthma. However, the intrinsic regulation of B cell function in this context is not fully understood. Here we investigated the role of Ssu72 phosphatase expressed in B cells during house dust mite (HDM)-induced asthma. Ssu72 expression was significantly increased in B cells stimulated with anti-CD40 and IL-4, as well as in B cells infiltrating the lungs of HDM-induced asthmatic mice. Notably, mice with B cell-specific deletion of Ssu72 exhibited considerably milder lung inflammation and lower serum levels of IgG1 and IgE compared to wild-type mice in HDM-induced asthma. In addition, the absence of Ssu72 in B cells resulted in a reduction in the number of IgG1- and IgE class-switched B cells and a decrease in soluble CD23 production, which correlated with lower expression of a disintegrin and metalloproteinase domain-containing protein 10 (ADAM10) and matrix metalloproteinase 9 (MMP9). Mechanistically, our findings suggest that Ssu72 deficiency may dysregulate the AMPK–mTORC1 balance through hyperphosphorylation of AMPKα. In adoptive transfer experiments, Ssu72-deficient B cells were less effective than wild-type B cells in promoting the effector phase of HDM-induced asthma in B cell-deficient hosts following HDM sensitization. Furthermore, B cell-specific deletion of Ssu72 led to the reduced production of IL-4 and IL-13 by CD4⁺ T cells and diminished MHC class II upregulation in lymph nodes and lung B cells during HDM-induced asthma. These findings identify B cell Ssu72 as a crucial regulator of B cell activation and function in allergic asthma.

## Ssu72 deficiency reduces asthma severity in mice

Allergic asthma involves B cells producing immunoglobulin E (IgE), which triggers allergic responses. This study identifies Ssu72 phosphatase as a novel regulator of B cell function in asthma, addressing the less-explored intrinsic mechanisms of IgE production. Researchers used B cell-specific Ssu72-knockout mice to investigate Ssu72’s role, revealing that its absence reduces asthmatic inflammation and IgE levels. The study found that Ssu72 deficiency disrupts the AMPK–mTORC1 signaling pathway, leading to impaired B cell proliferation and reduced T_H2_ immunity. This disruption also affects CD23 shedding, a process crucial for IgE regulation. These findings highlight Ssu72’s potential as a therapeutic target for asthma, offering a new avenue for treatment without compromising systemic immunity. Future research could explore Ssu72’s broader implications in immune regulation and its potential in other allergic conditions. This summary was initially drafted using artificial intelligence, then revised and fact-checked by the author.

## Introduction

B cells play a critical role in the development of allergic asthma by producing antibodies. Allergen-specific immunoglobulin E (IgE), produced exclusively by B cells, is the central mediator of the allergic response^1^. Upon re-exposure to allergens, these IgE antibodies bind to mast cells and basophils, leading to their activation and degranulation. This process is responsible for the pathology of allergic asthma and its clinical symptoms^2^. In addition, T helper 2 (T_H2_) cells that have been recruited to sites of allergenic entrance produce cytokines, such as IL-4, IL-5 and IL-13, which promote local IgE production, eosinophilia, airway hyperresponsiveness and mucus hypersecretion^3,4^.

The production of IgE can be regulated at multiple points, including class switch recombination (CSR) to IgE, the survival of IgE^+^ antibody-secreting cells and CD23-mediated regulation. CSR occurs in germinal centers (GCs) and requires assistance from T follicular helper (T_FH_) cells^5–7^. The isotype specificity of CSR is primarily determined by the production of germline transcripts, a process that is influenced by cytokines^8^. In the context of allergic asthma, T_H2_ cytokines promote CSR to IgE switching^9–11^. Notably, the production of IgE is limited by a shorter life span of IgE^+^ B cells than other isotype-switched B cells^12–14^. As a result, short-lived IgE^+^ antibody-secreting cells produce secretory IgE for only a brief period. In addition, this production is regulated by CD23 (FcεRII), a low-affinity IgE receptor on B cells^15^. Membrane-bound CD23 (mCD23) has been reported to negatively regulate IgE production, while soluble CD23 (sCD23) is believed to enhance it^16–18^. While the regulation of IgE production by B cell-extrinsic factors, such as IL-4, has been extensively studied, there is comparatively less understanding of the B cell-intrinsic molecular mechanisms that control IgE production. Identifying novel intrinsic regulators of B cell function is crucial for developing new strategies to suppress IgE production in allergic asthma.

The metabolic program has emerged as a critical regulator of B cell function by governing energy storage and the biosynthetic demands of activation, proliferation, differentiation and antibody production^19^. Adenosine monophosphate-activated protein kinase (AMPK) is a conserved serine/threonine kinase that acts as an energy sensor to restore cellular energy homeostasis. The activation of AMPK promotes fatty acid oxidation, autophagy and mitochondrial biogenesis while inhibiting the synthesis of proteins and lipids by triggering multiple downstream signaling pathways^20^. Mammalian target of rapamycin complex 1 (mTORC1) is a key target negatively regulated by AMPK. Several studies indicate that AMPK–mTORC1 signaling plays a crucial role in regulating effector function, the generation of memory cells and survival in T cells^21–23^. More recently, AMPK has been found to support the persistence of memory B cells, which is essential for robust recall humoral responses and the antibody production of plasma cells by regulating mTORC1 activity^24^. Moreover, AMPK activity can be regulated independently of energy stress in B cells, as AMPK phosphorylation increases after stimulation with anti-CD40 and IL-4^25^. This suggests a potential regulatory role for AMPK in antibody production. However, whether it regulates IgE production in B cells during allergic asthma remains uninvestigated.

Ssu72 is a dual protein phosphatase expressed in a tissue-specific manner^26–28^. Recently, Ssu72 has emerged as a receptor signaling-responsive phosphatase in immune cells, regulating their function, maturation and development^29–31^. Ssu72 was found to play a role in the fine-tuning of GM-CSF receptor signaling through binding to the GM-CSF receptor β chain, which regulates its phosphorylation in alveolar macrophages^30^. Moreover, Ssu72 binds to and regulates the phosphorylation of ZAP-70 and PLCγ1 in T cell receptor signaling^29,31^, suggesting an important role in T cells. However, the function of Ssu72 in B cells has not been thoroughly investigated.

In this study, we discovered that Ssu72 phosphatase levels were elevated in B cells following stimulation with anti-CD40, a combination of anti-IgM and anti-CD40, and IL-4, as well as in house dust mite (HDM)-induced asthma. Notably, B cell-specific Ssu72-knockout <8 mice exhibited reduced asthmatic inflammation, along with lower serum levels of IgG1 and IgE. Furthermore, the absence of Ssu72 in B cells impaired CD23 shedding, which was linked to decreased levels of a disintegrin and metalloproteinase domain-containing protein 10 (ADAM10) and matrix metalloproteinase 9 (MMP9). Mechanistically, Ssu72 deficiency disrupts the AMPK–mTORC1 signaling pathway by increasing the phosphorylation of AMPKα. In addition, this B cell defect results in reduced T_H2_ immunity in HDM-induced asthma.

Our findings reveal that Ssu72 serves as a novel regulator of B cell function and highlight its potential as a therapeutic target in asthma.

## Materials and methods

### Mice

The Ssu72-floxed *Ssu72*^*f/f*^ mouse strain, as described previously, was used in the experiment^27^. CD19-Cre mice were obtained from the Jackson Laboratory and mated in-house with *Ssu72*^*f/f*^ (wild-type, WT) mice to generate B cell-specific Ssu72-knockout mice (*CD19-Cre*; *Ssu72*^*f/f*^, Ssu72-BKO). μMT mice, which are deficient in B cells, were also purchased from the Jackson Laboratory and maintained in the animal facility. Both mouse strains were kept on a C57BL/6 background. Female mice aged 8–10 weeks were used in all experiments to minimize sex-related variability and avoid stress associated with male aggression.

### Antibodies

All antibodies used in this study are listed in Supplementary Table 1.

### B cells isolation and stimulation

Mouse spleens and mediastinal lymph nodes (mLNs) were isolated from WT and Ssu72-BKO mice, minced and passed through a 70-μm strainer (Sartorius) to obtain single-cell suspensions. The cells were suspended in R10 medium, which consists of RPMI-1640 (CM059-050, GenDEPOT) supplemented with 10% FBS (16000044, Gibco) and 1x Antibiotic Antimycotic (LS203-01, Welgene). Red blood cells were lysed using red blood cell lysis buffer (eBioscience). B cells were pre-enriched using the Magnisort Mouse B Cell Enrichment Kit (eBioscience), following the manufacturer’s instructions.

For B cell stimulation, isolated B cells were resuspended at 1 × 10^6^ cells/ml in B cell medium (R10 medium supplemented with 50 μM 2-mercaptoethanol and 1 mM sodium pyruvate). The cells were stimulated for 24 h at 37 °C with goat F(ab′)2 anti-mouse IgM (1 μg/ml, SouthernBiotech), purified hamster anti-mouse CD40 (HM40-3) (1 μg/ml, BD Biosciences), recombinant murine IFN-γ (25 ng/ml), recombinant murine IL-4 (25 ng/ml) or recombinant human TGF-β1 (20 ng/ml), all from Peprotech.

For the in vitro sCD23 release assay, isolated B cells from WT and Ssu72-BKO mice were incubated in B cell medium supplemented with lipopolysaccharide (LPS; 10 μg/ml), IL-4 (25 ng/ml) or LPS + IL-4 (10 μg/ml for LPS and 25 ng/ml for IL-4). The cells were incubated for 2 days at 37 °C and then harvested.

For in vitro IgE production, isolated B cells from WT and Ssu72-BKO mice were incubated in B cell medium supplemented with LPS (10 μg/ml), IL-4 (40 ng/ml) or LPS + IL-4 (10 μg/ml for LPS and 40 ng/ml for IL-4). The cells were incubated for the indicated days and then harvested. Supernatants were collected for enzyme-linked immunosorbent assay (ELISA) and stored at −80°C. Cell pellets were collected for fluorescence-activated single-cell sorting (FACS), reverse-transcription quantitative PCR (RT–qPCR) and western blot analysis.

### Generation of HDM-induced asthma model

WT and Ssu72-BKO mice were lightly anesthetized with isoflurane. To induce asthma, HDM extract (Greer Laboratories) was administered intranasally (i.n.) using a 10-µl pipette. Mice were sensitized with 1 μg of HDM in 10 μL i.n. once daily from days 0 to 2. After a 7-day rest period, mice were challenged with 3 μg of HDM in 10 μL i.n. five times on days 9 to 13. Mice in the control group received 10 μL of PBS i.n. following the same administration schedule as the HDM group. At 3 days after the last administration, serum samples were collected from the tail vein of each group, and the animals were euthanized for analysis. For WT and Ssu72-BKO mice, bronchoalveolar lavage fluid (BALF) samples were obtained by washing the lungs twice with 1 ml of PBS. For μMT mice, BALF samples were obtained by washing the lungs three times with 1 ml of PBS. BALF samples were centrifuged at 400*g* for 7 min at 4 °C. Total cell numbers were determined using a hemocytometer. Supernatant samples were collected for ELISA, and pellets were collected for FACS.

### Immunization of T cell-dependent and independent antigen

For T-dependent immune responses, mice were immunized with 100 μg 4-hydroxy-3-nitrophenylacetyl–keyhole limpet hemocyanin (NP-KLH; Biosearch Technologies) in alum (1:1) through intraperitoneal injection. For T-independent immune responses, mice were immunized intraperitoneally with 100 μg NP-LPS (Biosearch Technologies) or 100 μg NP-Ficoll (Biosearch Technologies). Before and 7, 14 and 21 days after immunization, serum samples were collected from the tail vein of each group. The mice were euthanized for analysis on day 21.

### Adoptive transfer

B cells were isolated from naive WT and Ssu72-BKO mice as described above, and a total of 5 × 10^6^ cells were adoptively transferred into sensitized μMT recipient mice intravenously, 2 days before the low-dose HDM challenge.

### Histopathological, immunofluorescence and immunohistochemical analysis

Mice lungs were fixed in formalin overnight at room temperature. Paraffin-embedded lung tissue blocks were prepared using standard methods and sectioned at 5–8-μm thickness. The sections were stained with hematoxylin and eosin (H&E), and lung inflammation was scored as previously described^32^.

For immunofluorescence staining, sections were deparaffinized through heating, hydration and antigen retrieval in citrate buffer. They were then blocked with 5% goat serum and stained with primary antibodies overnight at 4 °C, followed by incubation with secondary antibodies for 1 h at room temperature. The sections were mounted using DAPI-containing mounting medium (H-1200, Vector Laboratories).

For immunohistochemistry, sections underwent a similar deparaffinization process, followed by blocking with 5% goat serum. Primary antibodies were applied overnight at 4 °C, and the sections were incubated with an avidin–biotin–horseradish peroxidase (HRP) complex (Vectastain Elite ABS kit, Vector Laboratories). Peroxidase activity was visualized using a 3,3′-diaminobenzidine substrate kit (Vector Laboratories), after which the sections were counterstained with hematoxylin, dehydrated and mounted with Epredia Mounting Medium (Fisher Scientific). Images of the stained cells were captured using an Axio Imager M1 microscope (ZEISS) and processed with ZEISS ZEN microscope software. Positive areas were quantified using ImageJ.

### Flow cytometry

BALF cells, splenocytes and lymphocytes were washed with FACS buffer (0.5% FBS, 0.09% NaN3 in 1× PBS). The single-cell suspensions were stained on ice for 20 min with Fixable Viability Dye eFluor 780 to distinguish live from dead cells and with anti-CD16/CD32 to minimize non-specific antibody binding. For surface marker staining, the suspensions were incubated with fluorescent-conjugated antibodies for 30–60 min at 4 °C. Following staining, the cells were fixed using an Intracellular Fixation Buffer (eBioscience), in accordance with the manufacturer’s instructions. Intracellular IgE staining was performed as previously described^33–35^. In brief, the single-cell suspensions were treated for 1 min with an acid buffer to remove cytophilic IgE, followed by neutralization, fixation and permeabilization. Intracellular IgE staining was then performed using the FoxP3/Transcription Factor Staining Buffer Set Kit (eBioscience). To perform intracellular cytokine staining, the cells were resuspended in RPMI supplemented with 10% FBS, containing 50 ng/ml phorbol myristate acetate (PMA; Sigma-Aldrich), 500 ng/ml ionomycin (Cell Signaling), and 1 μl/ml of brefeldin A (GolgiPlug; BD Biosciences), and incubated in a 5% CO_2_ atmosphere for 5 h at 37 °C. Following surface staining, intracellular staining was conducted using a FoxP3/Transcription Factor Staining Buffer Set Kit (eBioscience), following the manufacturer’s guidelines. Afterward, the stained cells were washed with FACS buffer and analyzed using a BD FACS Canto II (BD Biosciences) and FlowJo software (FlowJo). Flow cytometry results were obtained by applying live cell gating and excluding doublets on the basis of forward scatter and side scatter parameters.

### RNA isolation and RT–qPCR

Total RNA was extracted from isolated B cells using an RNeasy Plus Mini Kit (QIAGEN), and complementary DNA (cDNA) was synthesized with a High-Capacity cDNA Reverse Transcription Kit (Thermo Fisher Scientific) using random primers. The cDNA was then subjected to PCR amplification with 2× PCR Taq MasterMix (Abcam), followed by RT–qPCR performed in triplicate using the SYBR Green Chemistry Kit (Applied Biosystems). Gene expressions were analyzed using the QuantStudio 6 Flex Real-Time PCR System (Fisher Scientific), and relative gene expressions were calculated using the comparative Ct (2^−∆∆Ct^) method. Average gene expression levels were normalized to *GAPDH* (glyceraldehyde-3-phosphate dehydrogenase) expression. The primers used were as follows: *Ssu72*, forward 5′-TCA AAG CAT GAT TGA GAG CAG CAG-3′, reverse 5′-GTG ATA GGC AAG CAG GTG TGA G-3′; *MMP9*, forward 5′-GCT GAC TAC GAT AAG GAC GGC A-3′, reverse 5′-TAG TGG TGC AGG CAG AGT AGG A-3′; *GAPDH*, forward 5′-CAT CTT CTT GTG CAG TGC CA-3′, reverse 5′-CGG CCA AAT CCG TTC AC-3′.

### ELISA

Serum NP-specific antibodies were tested by ELISA. In brief, 96-well plates were coated with NP_32_-BSA (Biosearch Technologies), followed by incubation with diluted serum samples and developed with HRP-conjugated mouse-specific anti-IgM and total IgG (SouthernBiotech). The HDM-specific IgGs were performed as previously described^36^, using 5 μg/ml HDM to coat for specific IgGs. Mouse IgE and IgG1 Uncoated ELISA Kits (Invitrogen) and Mouse CD23 ELISA Kit (Arigo Biolaboratories) were utilized according to the manufacturer’s instructions. After the final wash, TMB substrate (GeneDEPOT) was added to each well, and the color development was halted with Stop Solution for TMB Substrates (Invitrogen). Absorbance at 450 nm was measured using an xMark Microplate Spectrophotometer. Concentrations were calculated using a standard curve.

### Immunoblotting

The protein concentrations in lysates were determined using the Bradford method. Protein samples were boiled for 10 min in SDS sample buffer, then the denatured proteins were separated by SDS–PAGE and transferred to Clear Blot Membranes (ATTO). The membranes were blocked with Tris-buffered saline containing 0.1% Tween 20 (TBS-T) and 5% skimmed milk (BD Difco) for 30 min at room temperature while being rocked. They were then treated with primary antibodies in 5% skimmed milk or 5% BSA overnight at 4 °C with rocking. After washing with TBS-T four times, the membranes were incubated with HRP-conjugated secondary antibodies in 5% skimmed milk for 1.5 h at room temperature while being rocked. Proteins were detected using ECL Western blotting detection reagents (AbFrontier) and exposed to Medical X-ray film blue (Agfa-Gevaert N.V.).

### IP assay and Co-IP

Ramos cells were lysed using immunoprecipitation (IP) lysis buffer (20 mM Tris–HCl, pH 7.4, 150 mM NaCl; 1% Triton X-100; 1 mM EDTA; and 1 mM EGTA), supplemented with 1 mM phenylmethylsulfonyl fluoride (PMSF), 1 mM β-glycerophosphate and a 1× protein inhibitor cocktail. The lysate was centrifuged at 15,000*g* at 4 °C for 20 min. A 40-μg sample of the supernatant was retained as input, while 2,000 μg of total protein was mixed with either an anti-Ssu72 antibody or normal rabbit IgG as a negative control and rotated overnight at 4 °C. Following this, the mixture was rotated with 20 μl of a 50% slurry of protein A/G PLUS-Agarose (Santa Cruz Biotechnology) for 3 h at 4 °C. Immunoprecipitated proteins were then washed three times with lysis buffer and eluted with SDS sample buffer.

For the co-immunoprecipitation (Co-IP) assay, HEK293T cells were transfected with Myc-tagged Ssu72 (Myc-Ssu72), and AMPKα1 plasmids using linear polyethylenimine (PEI). When the cell reached 70–80% confluence, a transfection mixture containing DNA and PEI at a DNA:PEI ratio of 1:3 in Opti-MEM was added to the cells after mixing for 15 min at room temperature, followed by incubation for 36 h. A procedure similar to the IP assay was performed, with an anti-Myc antibody incubated with the proteins at 4 °C overnight.

### R2 gene expression analysis

The data from the analyses and comparison of gene expression in activated human B cells were obtained from the R2: Genomics Analysis and Visualization Platform (http://r2.amc.nl). Scatter plots illustrating the correlation between genes were generated, with the Spearman correlation coefficient (*r*) and *P* values calculated using GraphPad Prism.

### Study approval

All animal experiments were conducted in accordance with the guidelines of the Institutional Animal Care and Use Committee (IACUC) at Sungkyunkwan University School of Medicine. The Sungkyunkwan University School of Medicine is accredited by the Association for Assessment and Accreditation of Laboratory Animal Care International and complies with the guidelines issued by the Institute of Laboratory Animal Resources.

### Statistical analysis

*P* values were calculated using Student’s *t*-test, one-way analysis of variance (ANOVA) followed by Dunnett’s multiple comparison test or two-way ANOVA followed by Bonferroni’s post hoc test (GraphPad Prism). A *P* value of less than 0.05 was considered significant. Data are presented as mean ± s.d.

## Results

### The expression of Ssu72 is upregulated in B cells from HDM-induced asthma model

**Fig. 1:**
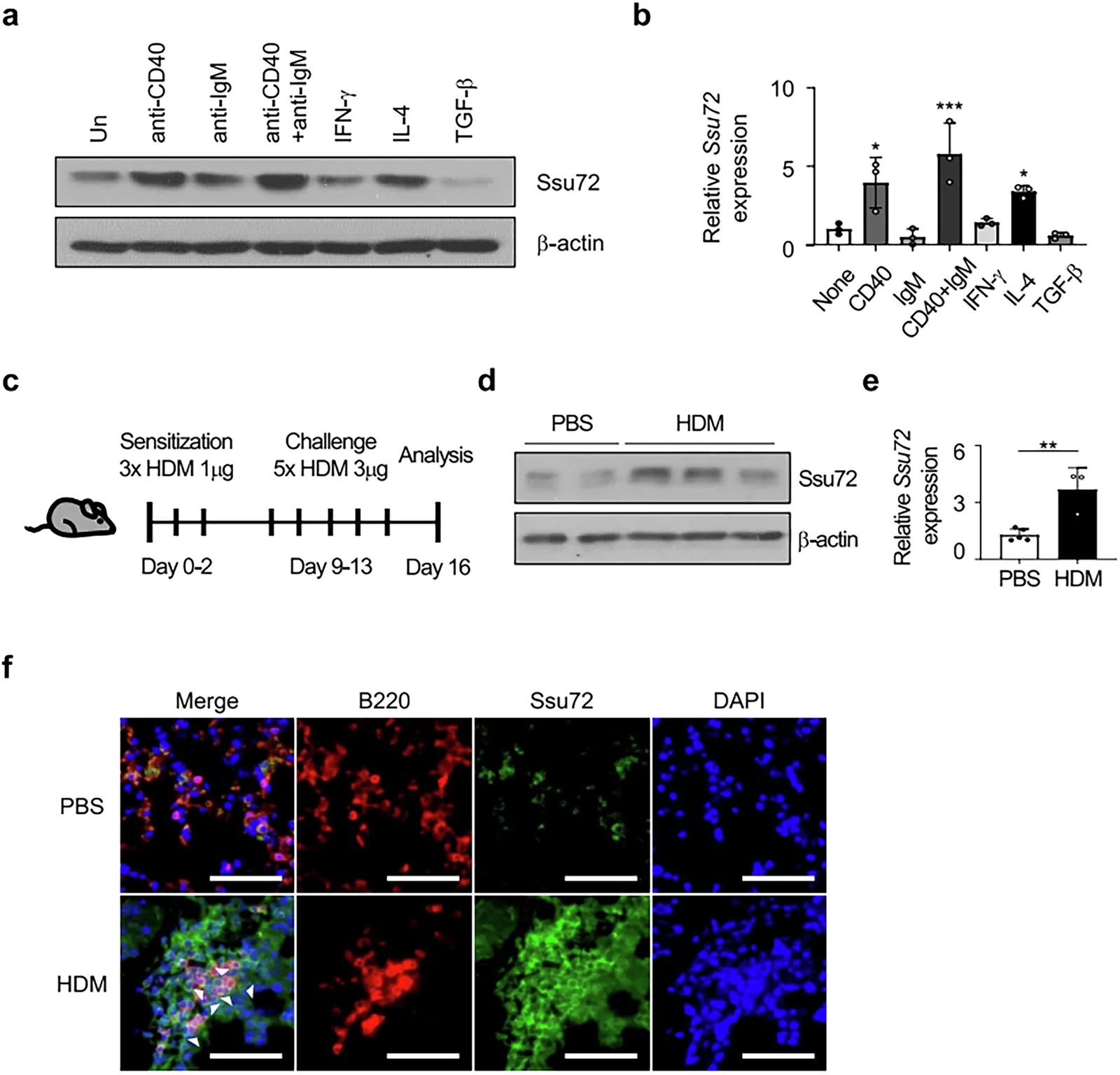
The expression level of Ssu72 in the T_H2_ stimulation and asthma model. **a** Immunoblot analysis of Ssu72 expression in isolated B cells after stimulation for 24 h. Cells were treated with anti-CD40 (1 μg/ml), anti-IgM (1 μg/ml) or anti-CD40 + anti-IgM (1 μg/ml each) and exposed to Th1 cytokine IFN-ɣ (25 ng/ml), T_H2_ cytokine IL-4 (25 ng/ml) and T_reg_ cytokine TGF-β (20 ng/ml). β-actin served as the loading control. **b** An RT–qPCR analysis of *Ssu72* mRNA expression was also performed on B cells following the same treatment for 24 h. Gene expression was normalized against *GAPDH* (*n* = 3 or 4 per group). **c** Schematic depicting the induction of HDM-induced asthma in WT mice. **d** Immunoblot analysis of Ssu72 expression in isolated splenic B cells from the PBS-injected control group and the HDM-induced asthma group. **e** RT–qPCR for *Ssu72* mRNA expression was determined using B cells from the spleens of the PBS-injected control group and the HDM-induced asthma group. Normalization was performed using *GAPDH* (*n* = 3–5 per group). **f** Immunofluorescence staining for B220 (red), Ssu72 (green) and DAPI (blue) in the lungs of PBS-injected controls and the HDM-induced asthma group. Scale bars, 50 μm. Data are presented as mean ± s.d. *P < 0.05, **P < 0.01, ***P < 0.001, Student’s *t*-test or one-way ANOVA followed by Dunnett’s multiple comparisons test.

To investigate the role of Ssu72 in B cells, we analyzed its potential association with various B cell receptor signaling pathways. Ssu72 was notably upregulated in response to anti-CD40, the combination of anti-IgM and anti-CD40, and the T_H2_ cytokine IL-4 (Fig. 1a, b). Given that CD40 and IL-4 are crucial for B cell activation and IgE class switching during allergic responses, we further examined Ssu72 expression in B cells within the context of allergic asthma. We used a low-dose HDM sensitization and challenge protocol to induce a murine model of allergic asthma (Fig. 1c). B cells were isolated from the spleens of PBS-injected control mice and those subjected to the HDM-induced asthma model. We found that both protein and mRNA levels of Ssu72 were significantly elevated in B cells from the HDM model compared to those from the control mice (Fig. 1d, e). In addition, lung tissues from HDM-induced asthmatic mice exhibited an increased population of B220^+^ B cells and heightened Ssu72 expression (Fig. 1f, white arrows), while B cells in the lungs of PBS-treated control mice showed low Ssu72 expression (Fig. 1f). Collectively, these findings suggest that Ssu72 is upregulated in activated B cells under T_H2_-polarizing conditions and in lung-infiltrating B cells in mice with HDM-induced asthma, indicating that Ssu72 may play an important role in B cell function.

### Ssu72 deficiency in B cells alleviates airway inflammation and reduces IgE levels in HDM-induced asthma

To explore the role of Ssu72 in B cell function, we conditionally deleted the *Ssu72* gene in B cells. This was achieved by breeding mice with a floxed *Ssu72* *gene* (*Ssu72*^*f/f*^, WT) with mice carrying *CD19-Cre*, resulting in the deletion of the Ssu72 gene specifically in CD19^+^ B cells. B cell-specific Ssu72-knockout mice (*CD19-Cre*; *Ssu72*^*f/f*,^ Ssu72-BKO) were generated (Supplementary Fig. 1a). A reduction in Ssu72 protein levels was observed specifically in Ssu72-BKO B cells, while no effect was noted in other organs (Supplementary Fig. 1b). Moreover, mRNA expression of *Ssu72* was significantly reduced in Ssu72-BKO B cells (Supplementary Fig. 1c).

Next, we evaluated the percentage and total number of B cells in the spleen. Both the percentage and total number of B cells in the spleen were comparable between WT and Ssu72-BKO mice (Supplementary Fig. 2a, b). However, there was a significant decrease in the percentage of T1 B cells (CD19^+^CD93^+^IgM^+^CD23^−^) and marginal zone B cells (CD19^+^CD21^+^CD23^−^), while the percentage of T2/3 B cells (CD19^+^CD93^+^IgM^+/−^CD23^+^) and follicular B cells (CD19^+^CD21^int^CD23^+^) exhibited an increase in Ssu72-BKO mice compared to WT mice (Supplementary Fig. 2c, d). In addition, the expression of CD23 on B cells and CD23^+^ population in overall B cells were increased in Ssu72-BKO mice compared to WT mice (Supplementary Fig. 2e, f). To exclude the potential impact of increased CD23 expression on B cells, we used an alternative gating strategy without CD23 marker. Interestingly, we found the population of follicular B (CD19^+^IgM^lo^IgD^hi^), T2 B (CD19^+^IgM^hi^IgD^hi^), T1 (CD19^+^IgM^hi^IgD^lo^CD21^−^) and marginal zone B (CD19^+^IgM^hi^IgD^lo^CD21^+^) cells remained unchanged between WT and Ssu72-BKO mice (Supplementary Fig. 2g), suggesting that the deletion of Ssu72 in B cells had no significant impact on B cell development. As one of the primary functions of B cells is to protect the host through antibody production, it was crucial to examine the impact of Ssu72 deficiency in B cell function. The Ssu72-BKO mice produced the comparable amount of NP-specific IgM and IgG in the sera compared to WT mice, when the mice were immunized with NP-LPS, NP-Ficoll and NP-KLH (Supplementary Fig. 3a, b). Moreover, there was no difference in GC formation and the population of plasma cells between WT and Ssu72-BKO mice (Supplementary Fig. 3c, d). These data suggest that the deficiency of Ssu72 in B cells exhibits no impact on antibody secretion and differentiation despite CD23 surface overexpression.

**Fig. 2:**
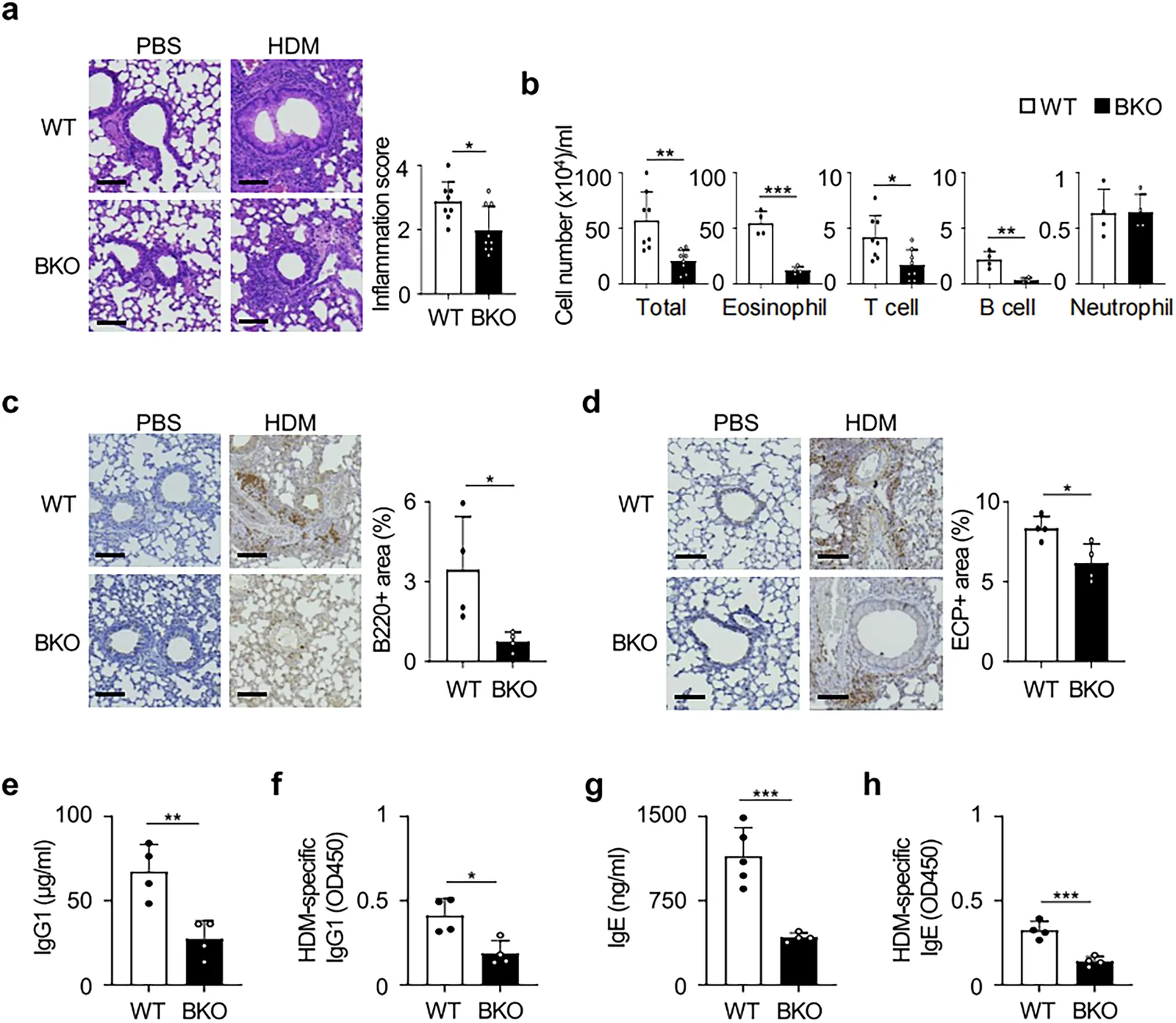
The effect of B cell-intrinsic Ssu72 on asthma airway inflammation in HDM-induced asthma. **a** Representative H&E staining of lung sections from WT and Ssu72-BKO mice after PBS or HDM injection. Scale bar, 100 μm. Lung inflammatory scores were calculated (*n* = 8 or 9 per group). **b** Numbers of total BALF cells, neutrophils, eosinophils, T cells and B cells in WT and Ssu72-BKO mice with HDM-induced asthma (*n* = 4–8 per group). **c** B220 immunohistochemistry of PBS-injected and HDM-injected WT and Ssu72-BKO mouse lung sections. Scale bars, 100 μm. The quantification of B220^+^ area in the lungs of the HDM-induced asthma model (*n* = 4 per group). **d** ECP immunohistochemistry of PBS-injected and HDM-injected WT and Ssu72-BKO mouse lung sections. Scale bars, 100 μm. The quantification of ECP^+^ areas in the lungs of the HDM-induced asthma model (*n* = 4 per group). **e**, **f** Total serum IgG1 (**e**) and HDM-specific IgG1 (**f**) were measured by ELISA (*n* = 4 per group). **g**, **h** Total serum IgE (**g**) and HDM-specific IgE (**h**) were measured by ELSIA (*n* = 4 or 5 per group). Data are presented as mean ± s.d. *P < 0.05, **P < 0.01, ***P < 0.001, Student’s *t*-test.

To explore the potential role of B cell-expressed Ssu72 in the development of allergic asthma, we established HDM-induced asthma in WT and Ssu72-BKO mice. The results showed that Ssu72-BKO mice exhibited fewer inflammatory cells in their asthmatic lungs compared to WT mice, along with lower inflammation scores (Fig. 2a). We further evaluated cellular infiltrates in the BALF, with the gating strategy detailed in Supplementary Fig. 4. Notably, Ssu72-BKO mice with HDM-induced asthma had significantly reduced total cell counts in the BALF, including fewer eosinophils, T cells and B cells compared to WT mice (Fig. 2b). In addition, immunohistochemical staining for B220 (a B cell marker) and eosinophil cationic protein (ECP, an eosinophil marker) confirmed the reduced infiltration of B cells and eosinophils in lung tissues (Fig. 2c, d). Serum levels of total and HDM-specific IgE and IgG1 were significantly lower in asthmatic Ssu72-BKO mice compared to their WT counterparts (Fig. 2e–h). By contrast, the levels of HDM-specific IgM, IgG2b and IgG3 were similar between the two groups (Supplementary Fig. 5a–c), suggesting that B cell-specific Ssu72 deficiency preferentially affects IgG1 and IgE antibody responses in HDM-induced asthma. These findings indicate that the upregulation of Ssu72 in activated B cells contributes to lung inflammation and high serum IgE levels in HDM-induced asthma.

### Loss of Ssu72 reduces class-switched IgG1^+^ and IgE^+^ in B cells

**Fig. 3:**
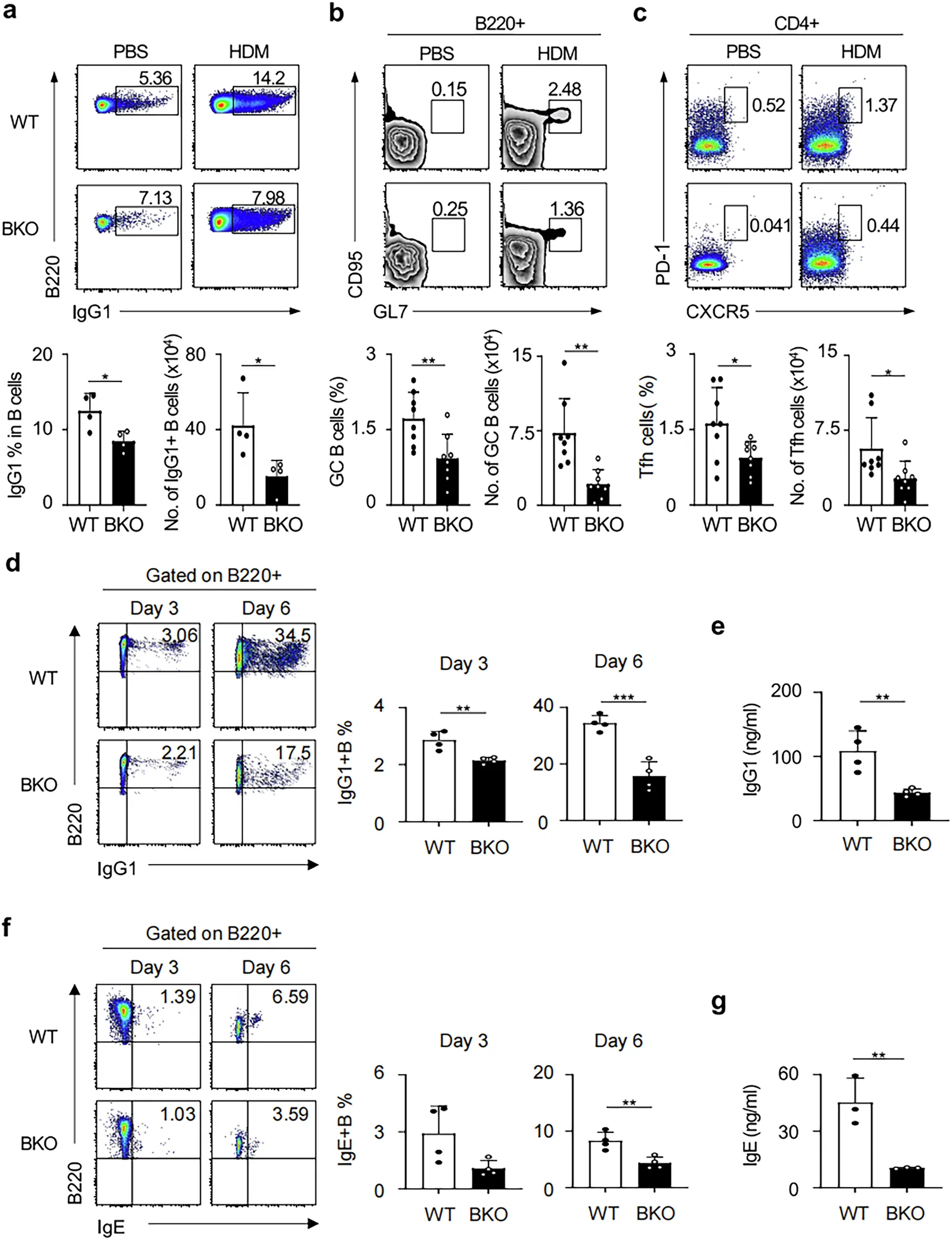
The loss of Ssu72 reduces the numbers of class-switched IgG1^+^ and IgE^+^ B cells. **a** Representative flow cytometry plots of IgG1^+^ B cells in mLNs in indicated mice. Percentages and numbers of IgG1^+^ B cells (*n* = 4 per group). **b** Representative flow cytometry plots of GC B cell numbers (B220^+^CD95^+^GL7^+^) in the mLNs of indicated mice. Percentages and numbers of GC B cells (*n* = 8 per group). **c** Representative flow cytometry plots of T_FH_ cells (CD4^+^PD-1^+^CXCR5^+^) in the mLNs of indicated mice. Percentages and numbers of T_FH_ cells in CD4^+^ T cells (*n *= 8 per group). **d** Representative flow cytometry plots of IgG1^+^ B cells from WT and Ssu72-deficient B cells stimulated with LPS (10 μg/ml) plus IL-4 (40 ng/ml) for 3 and 6 days. Percentages of IgG1^+^ B cells in B cells at the indicated time point (*n* = 4 per group). **e** Levels of IgG1 in the supernatant after stimulation for 6 days (*n* = 4 per group). **f** Representative flow cytometry plots of intracellular IgE^+^ B cells from WT and Ssu72-deficient B cells stimulated with LPS plus IL-4 for 3 and 6 days. Percentages of IgE^+^ B cells in B cells at the indicated time points (*n* = 3 or 4 per group). **g** Levels of IgE in supernatant after stimulation for 6 days (*n* = 3 per group). Data are presented as mean ± s.d. *P < 0.05, **P < 0.01, ***P < 0.001, Student’s *t*-test.

Given that IgE-expressing cells can be generated through direct switching from IgM to IgE or through sequential CSR from IgG1 to IgE^37,38^, we hypothesized that the number of class-switched IgG1^+^ B cells would be reduced in Ssu72-BKO mice during HDM-induced asthma. First, we assessed the number of B cells in the mLNs. A slight decrease in the total number of B cells was observed in Ssu72-BKO mice compared to WT mice after HDM-induced asthma; however, this difference was not statistically significant (Supplementary Fig. 6a). By contrast, both the percentage and number of IgG1^+^ B cells were significantly decreased in Ssu72-BKO mice compared to WT mice following asthma induction (Fig. 3a). B cells undergo CSR to IgG1 and IgE with the assistance of T_FH_ cells within the GC^39,40^. To determine whether the decrease in IgG1^+^ B cells and the reduced levels of IgG1 and IgE are associated with altered GC formation and T_FH_ cells in HDM-induced asthma, we assessed the percentage and absolute number of GC B cells and T_FH_ cells in the mLN. Notably, both the percentage and number of GC B cells were significantly decreased in Ssu72-BKO mice after HDM-induced asthma compared to WT mice (Fig. 3b). In addition, the percentage and number of T_FH_ cells were also significantly reduced in Ssu72-BKO mice compared to WT mice following HDM-induced asthma (Fig. 3c), despite no significant decrease in total T cell numbers (Supplementary Fig. 6b). These observations suggest that Ssu72 deficiency in B cells leads to reduced populations of GC B and T_FH_ cells in HDM-induced asthma.

To investigate whether the deletion of Ssu72 in B cells results in intrinsic defects in IgE and IgG1 production, we stimulated purified B cells with IL-4 and LPS to induce CSR to IgE and IgG1 in vitro. Interestingly, the percentage of IgG1^+^ B cells was markedly reduced in Ssu72-deficient B cells compared to WT B cells after 3 and 6 days of stimulation (Fig. 3d). Furthermore, there was a significant reduction in the percentage of IgE^+^ B cells in Ssu72-deficient B cells compared to WT B cells after 6 days of stimulation (Fig. 3f). Moreover, the production of IgG1 and IgE was significantly lower in Ssu72-deficient B cells compared to WT B cells (Fig. 3e, g). However, there were no differences in the percentages of IgM^+^ B cells after 3 and 6 days of stimulation, and the levels of IgM secretion were also comparable (Supplementary Fig. 7a, b).

As isotype switching is closely associated with B cell proliferation^41^, we next compared the proliferative capacities and survival of Ssu72-deficient and WT B cells upon stimulation with a combination of IL-4 and LPS. Ssu72-deficient B cells proliferated significantly less, as determined by the percentage of Ki67^+^ B cells (Supplementary Fig. 8a), and exhibited a smaller proportion of live cells, as assessed by Annexin V^−^PI^−^ staining (Supplementary Fig. 8b). Early activation was also reduced in Ssu72-deficient B cells, as indicated by lower upregulations of CD69 and CD86 at 24–48 h (Supplementary Fig. 8c, d). Taken together, these findings suggest that Ssu72-deficient B cells have an intrinsic defect in IgG1 and IgE antibody responses, which is accompanied by impaired proliferation, survival, and activation.

### B cell-intrinsic Ssu72 is involved in the regulations of mCD23 expression and sCD23 generation

**Fig. 4:**
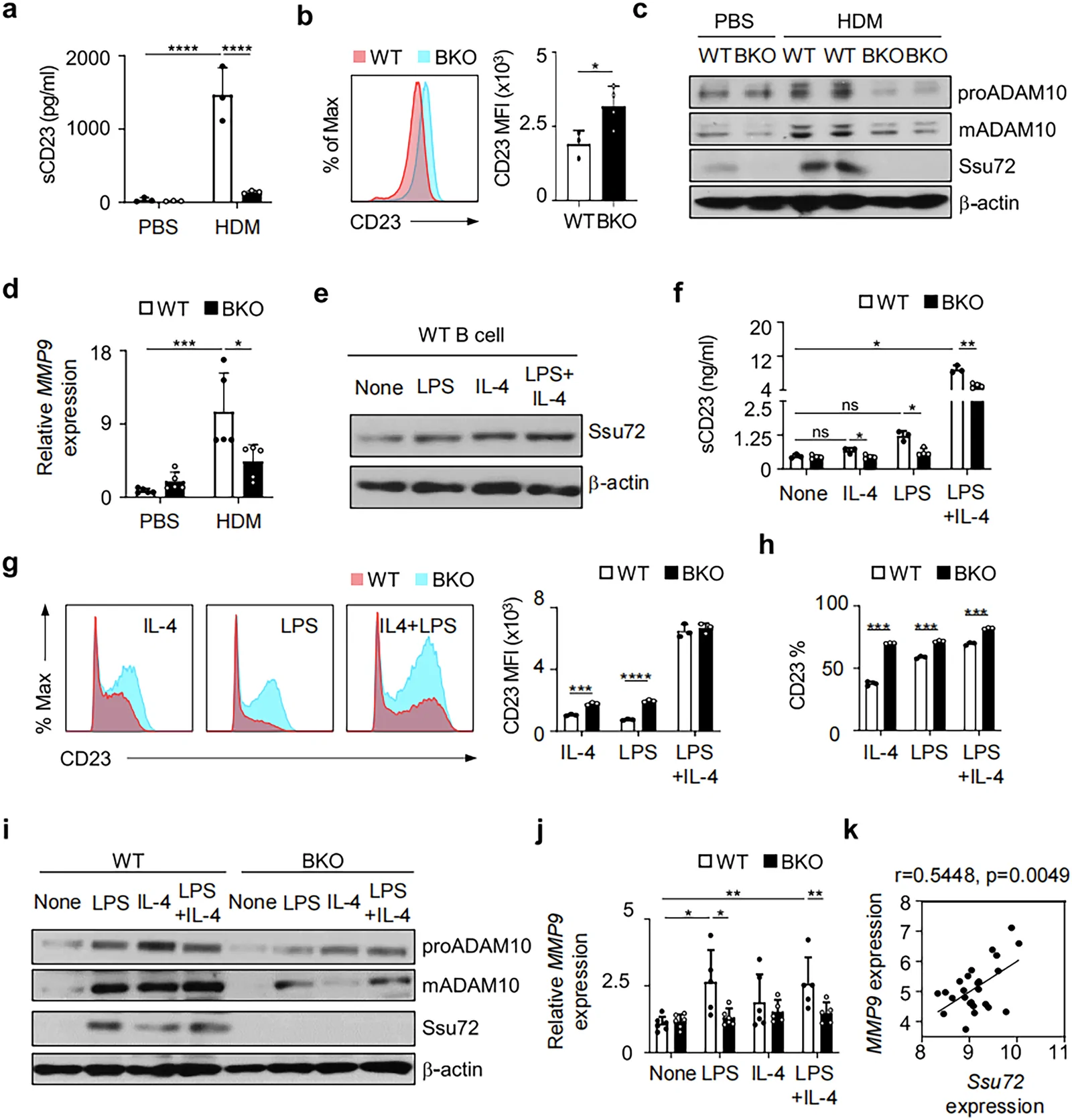
B cell-intrinsic Ssu72 regulates sCD23 generation in vivo and in vitro. **a** Levels of sCD23 in the sera of WT and Ssu72-BKO mice from the PBS-injected group and HDM-induced asthma group (*n* = 3 or 4 per group). **b** Representative histogram of the mean fluorescence intensities (MFIs) of CD23 on B cells and MFI values of the mLNs from WT and Ssu72-BKO mice in the HDM-induced asthma model group (*n* = 3 or 4 per group). **c** The protein level of proADAM10 and mADAM10 in B cells isolated from WT and Ssu72-BKO mice in the PBS-injected group and HDM-induced asthma model group. **d** RT–qPCR for *MMP9* mRNA expression was determined using B cells from PBS- and HDM-injected WT and Ssu72-BKO mice (*n *= 5 or 6 per group). **e** The protein level of Ssu72 in B cells, untreated or treated with IL-4 (25 ng/ml), LPS (10 μg/ml) or LPS + IL-4 for 24 h. **f** Levels of sCD23 in supernatants collected following stimulation for 48 h. B cells isolated from WT and Ssu72-BKO mice were untreated or treated with IL-4, LPS or LPS + IL-4 (*n* = 3 or 4 per group). **g** Representative histogram of CD23 expression and MFI values in stimulated B cells of WT and Ssu72-BKO mice (*n* = 3 per group). **h** Percentages of CD23^+^ B cells after stimulating B cells from WT and Ssu72-BKO mice (*n* = 3 per group). **i** Protein levels of proADAM10 and mADAM10 in stimulated B cells from WT and Ssu72-BKO mice. **j** The expression of *MMP9* mRNA levels in B cells after stimulation of LPS, IL-4 or LPS + IL-4 for 24 h. **k** Scattered plots showing the correlation of gene expression between *MMP9* and *Ssu72* in in vitro activated human B cells obtained from a public data set on the R2 platform (*n* = 25). The Spearman correlation coefficient (*r*) and associated *P* value were calculated using GraphPad Prism. Data are presented as mean ± s.d. ns, not significant. *P < 0.05, **P < 0.01, ***P < 0.001, ****P < 0.0001, Student’s *t*-test, two-way ANOVA followed by Bonferroni’s post hoc test.

CD23 is a low-affinity IgE receptor that inhibits IgE synthesis, while sCD23, produced by the cleavage of mCD23, enhances IgE synthesis^15,17,42^. We investigated whether the deletion of Ssu72 in B cells affects mCD23 expression and sCD23 generation, leading to reduced levels of IgE in HDM-induced asthma. We assessed serum levels of sCD23 in WT and Ssu72-BKO mice following PBS or HDM injection. A significant reduction in sCD23 levels was observed in Ssu72-BKO mice after HDM induction compared to WT mice (Fig. 4a). However, mCD23 levels were higher in mLN B cells from Ssu72-BKO mice than in WT mice after asthma induction (Fig. 4b), suggesting that Ssu72 in B cells may play a role in regulating CD23 during HDM-induced asthma.

Given that ADAM10 has been identified as a sheddase for mCD23, we next investigated whether the observed reduction in sCD23 was associated with ADAM10 levels. The protein levels of proADAM10 and mature ADAM10 (mADAM10) were assessed in B cells isolated from WT and Ssu72-BKO mice, both with and without asthma induction. After HDM administration, proADAM10 and mADAM10 levels were elevated in B cells from WT mice (Fig. 4c), suggesting that ADAM10 was induced during HDM-induced asthma. However, proADAM10 and mADAM10 levels were lower in B cells from Ssu72-BKO mice compared to those from WT mice with HDM-induced asthma (Fig. 4c). MMP9 has also been suggested to play a role in cleaving mCD23 to sCD23 on B cells^43^. We next compared *MMP9* mRNA levels in B cells from WT and BKO mice after HDM-induced asthma. A notable decrease in *MMP9* mRNA levels was observed in B cells from Ssu72-BKO mice compared to WT mice in the HDM-induced asthma model, while there was no difference in PBS-injected control mice (Fig. 4d).

To assess whether Ssu72 deficiency affects sCD23 generation in B cells intrinsically, we isolated splenic B cells from WT and Ssu72-BKO mice and incubated them with IL-4, LPS or a combination of both. The protein levels of Ssu72 showed an increase after stimulation (Fig. 4e). Ssu72-deficient B cells exhibited significantly reduced sCD23 levels compared to WT B cells under all stimulation conditions (Fig. 4f). By contrast, mCD23 expression was notably higher in Ssu72-deficient B cells following stimulation with either IL-4 or LPS (Fig. 4g). Moreover, the percentage of CD23^+^ cells was greater in Ssu72-deficient B cells than in WT B cells after stimulation with IL-4, LPS or both (Fig. 4h). Moreover, the levels of proADAM10 and mADAM10 were lower in Ssu72-deficient B cells compared to WT B cells after stimulation (Fig. 4i). The expression of *MMP9* mRNA was also lower in Ssu72-deficient B cells following LPS and LPS + IL-4 stimulation, while no significant differences were observed with IL-4 stimulation alone (Fig. 4j). This suggests that Ssu72 may regulate CD23 shedding through LPS-induced *MMP9* transcription. A further analysis of publicly available RNA sequencing data from activated human B cells revealed a positive correlation between Ssu72 and *MMP9* expression (Fig. 4k), which supports our findings. Overall, these results indicate that Ssu72 deficiency in B cells leads to impaired CD23 shedding and sustained higher mCD23 expression in both in vivo HDM-induced asthma and in vitro stimulations.

### B cell-intrinsic Ssu72 interacts with AMPKα

**Fig. 5:**
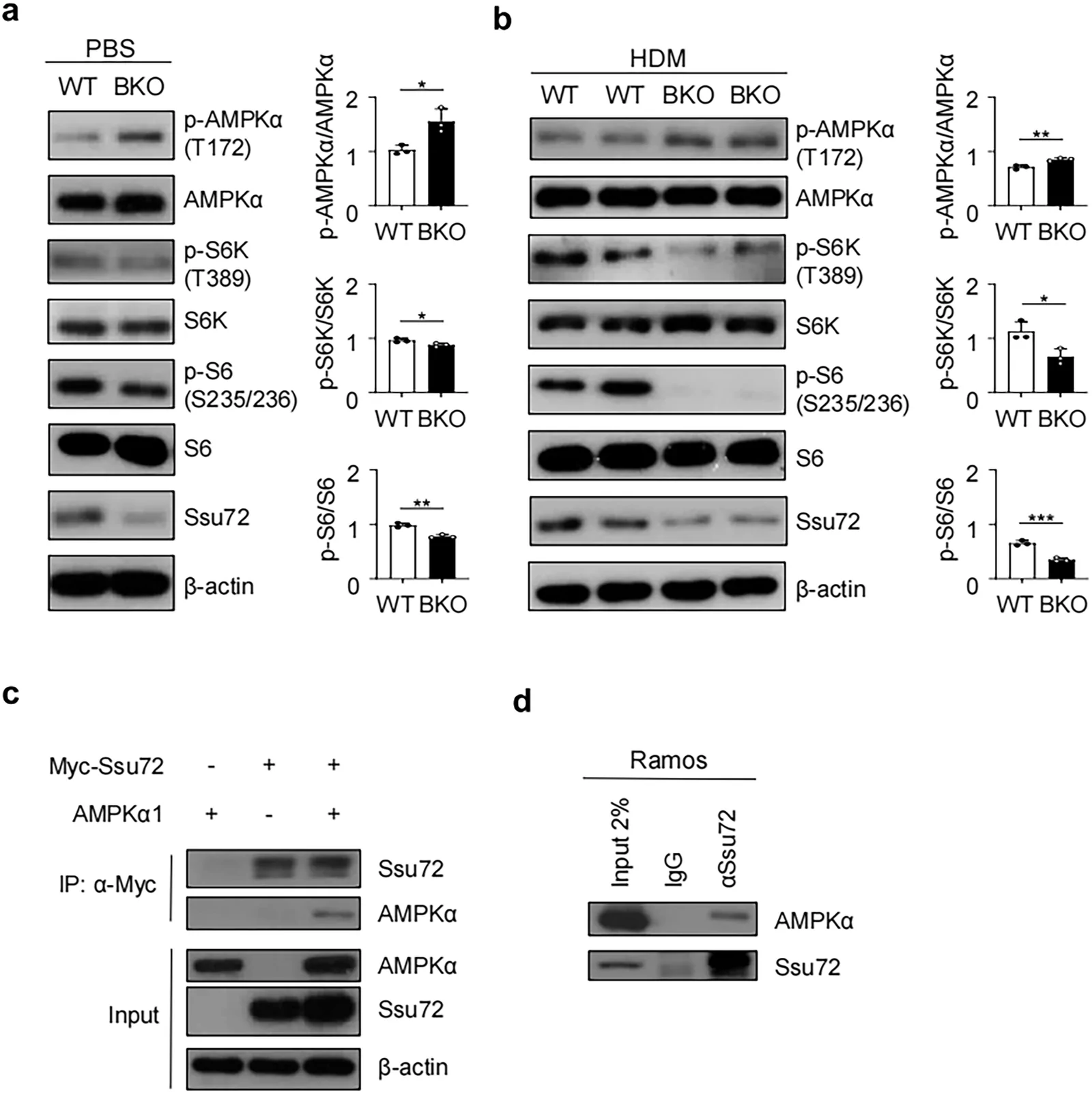
The loss of Ssu72 increases p-AMPKα levels in B cells. **a** Immunoblot of p-AMPKα, AMPKα, p-S6K, S6K, p-S6 and S6 in B cells isolated from WT and Ssu72-BKO mice in the PBS-injected group. Densitometric analysis of p-AMPKα/AMPKα, p-S6K/S6K and p-S6/S6 (*n* = 3 per group). **b** Immunoblot of p-AMPKα, AMPKα, p-S6K, S6K, p-S6 and S6 in B cells isolated from WT and Ssu72-BKO mice in the HDM-induced asthma model group. Densitometric analysis of p-AMPKα/AMPKα, p-S6K/S6K and p-S6/S6 (*n* = 3 per group). **c** Co-IP assays were performed using HEK293T cells transfected with expression vectors for Myc-tagged Ssu72 (Myc-Ssu72) and AMPKα1 plasmid. Cellular extracts were immunoprecipitated with an anti-Myc antibody. IP and the input were detected by western blot using indicated antibodies. **d** For the IP assay, Ramos cells were lysed and then immunoprecipitated with an anti-Ssu72 antibody, followed by immunoblotting with the indicated antibodies. Data are presented as mean ± s.d. *P < 0.05, **P < 0.01, ***P < 0.001, Student’s *t*-test.

To understand the underlying mechanism of the defect in B cell function caused by Ssu72 depletion, we examined the expression profiles of key marker molecules involved in signaling pathways related to asthma, using B cells isolated from WT and Ssu72-BKO mice. Notably, the expression of phosph-S6K^T389^ and phospho-S6^S235/S236^, which are the downstream targets of mTORC1, was significantly reduced in Ssu72-BKO B cells compared to WT B cells, particularly in asthmatic B cells (Fig. 5a, b). Because AMPK negatively regulates mTORC1 activity in antibody responses, we next assessed the expression levels of AMPK. Surprisingly, phosphorylation of AMPKα at Thr172 was sharply increased in B cells from Ssu72-BKO mice compared to WT mice, regardless of PBS or HDM induction (Fig. 5a, b). To determine the potential interaction between Ssu72 and AMPKα, we performed a Co-IP assay using cell lysates from 293T cells transfected with Myc-Ssu72 and AMPKα1 plasmids. Interestingly, we observed a complex formation between Ssu72 and AMPKα (Fig. 5c). Furthermore, the endogenous interaction between Ssu72 and AMPKα was also demonstrated in Ramos cells (Fig. 5d). Collectively, these findings suggest that Ssu72 can interact with AMPKα in B cells.

### Ssu72 deficiency in B cells attenuates airway inflammation in the effector phase of HDM-induced asthma

**Fig. 6:**
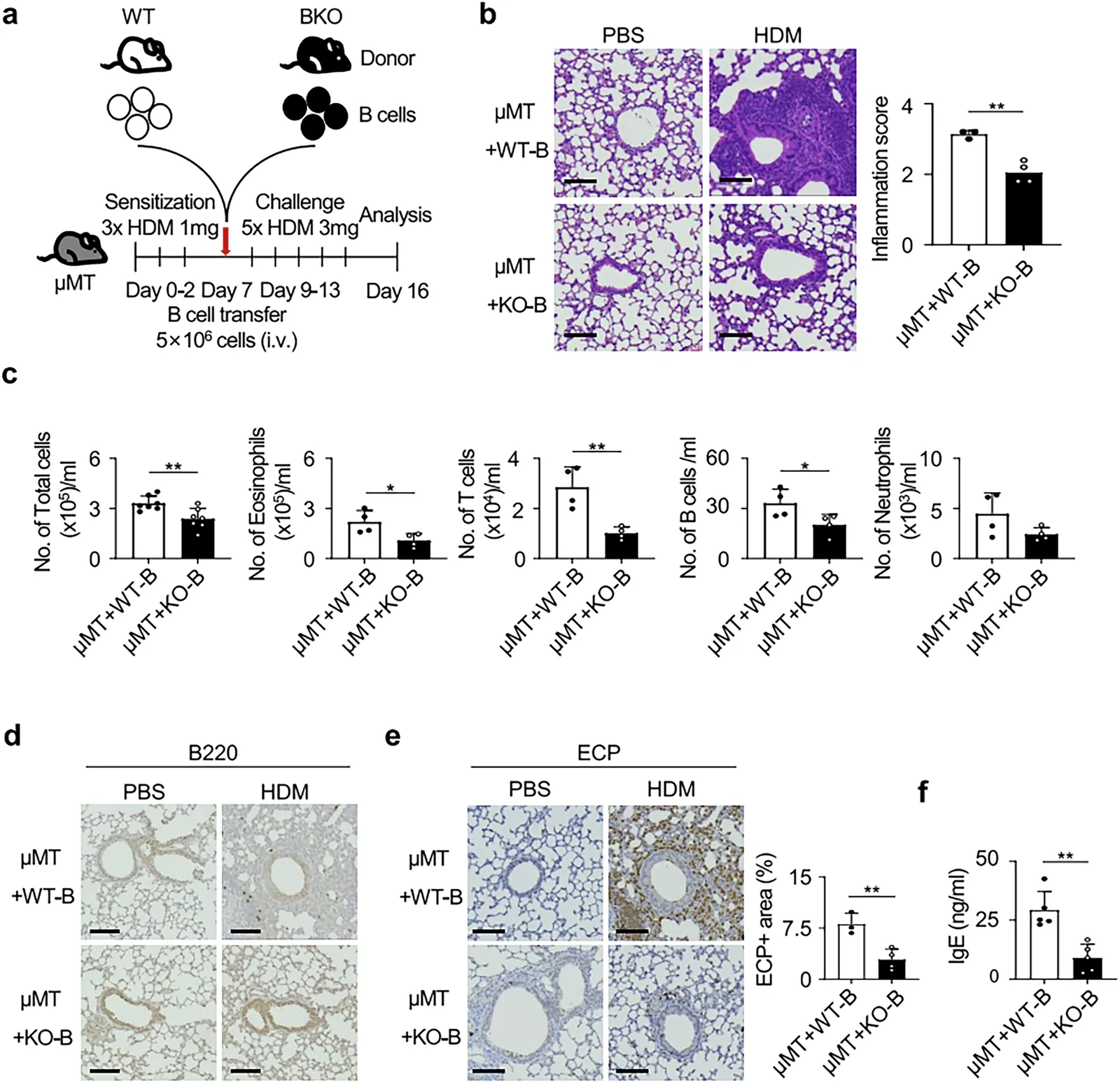
B cell-intrinsic Ssu72 is essential at the effector phase of allergic asthma. **a** Schematic diagram showing adoptive transfer protocol. μMT mice were sensitized with HDM 1 μg i.n. on days 0 to 2, and B cells (purity ≥95%) isolated from WT or Ssu72-BKO mice (5 × 10^6^ cells) were adoptively transferred intravenously into sensitized uMT mice 2 days before challenge. On days 9 to 13, μMT mice were challenged with HDM 3 μg per day. Analysis was performed on day 16. **b** Representative H&E lung sections of μMT mice receiving WT B cells or Ssu72-deficient B cells were examined after injection of PBS or HDM. Scale bars, 100 μm. Lung inflammatory scores were determined by histologically analyzing lung tissues (*n* = 3 or 4 per group). **c** Numbers of total BALF cells, neutrophils, eosinophils, T cells and B cells in μMT mice with HDM-induced asthma transferred with WT B cells or Ssu72-deficient B cells (*n* = 4–8 per group). **d** Representative B220 immunohistochemistry results of PBS-injected or HDM-challenged mice transferred with WT or Ssu72-deficient B cells. Scale bars, 100 μm. **e** Representative ECP immunohistochemistry results of PBS-injected or HDM-challenged mice transferred with WT or Ssu72-deficient B cells. Scale bars, 100 μm. Quantification of ECP^+^ areas in the lungs of the HDM-induced asthma model (*n* = 3 or 4 per group). **f** The total serum IgE was measured by ELSIA (*n* = 5 per group). Data are presented as mean ± s.d. *P < 0.05, **P < 0.01, ***P < 0.001, Student’s *t*-test.

To further explore the role of B cell Ssu72 in the effector phase of HDM-induced asthma, we transferred either WT or Ssu72-deficient B cells into previously sensitized B cell-deficient μMT mice, followed by HDM challenge (Fig. 6a). Successful engraftment of transferred B cells into μMT mice was observed in the mLNs and spleens, with no differences in the percentages or absolute numbers of B cells between mice that received WT B cells and those that received Ssu72-deficient B cells (Supplementary Fig. 9a–f). Notably, the transfer of Ssu72-deficient B cells resulted in reduced lung inflammation and lower inflammation scores (Fig. 6b). In addition, mice receiving Ssu72-deficient B cells exhibited significantly fewer total cells, eosinophils, T cells and B cells in the BALF compared to those receiving WT B cells (Fig. 6c). Immunohistochemistry for B220 and ECP on lung tissues further demonstrated decreased B cell and eosinophil recruitment in mice that received Ssu72-deficient B cells versus those that received WT B cells (Fig. 6d, e). Furthermore, IgE levels were significantly lower in mice transferred with Ssu72-deficient B cells compared to those with WT B cells (Fig. 6f). These findings suggest that Ssu72 deficiency in B cells reduces airway inflammation during the effector phase and indicates that B cell Ssu72 plays a role beyond lymphoid tissue in the context of asthmatic inflammation.

### Ssu72 deficiency in B cells alleviates T_H2_ immune response in HDM-induced asthma

**Fig. 7:**
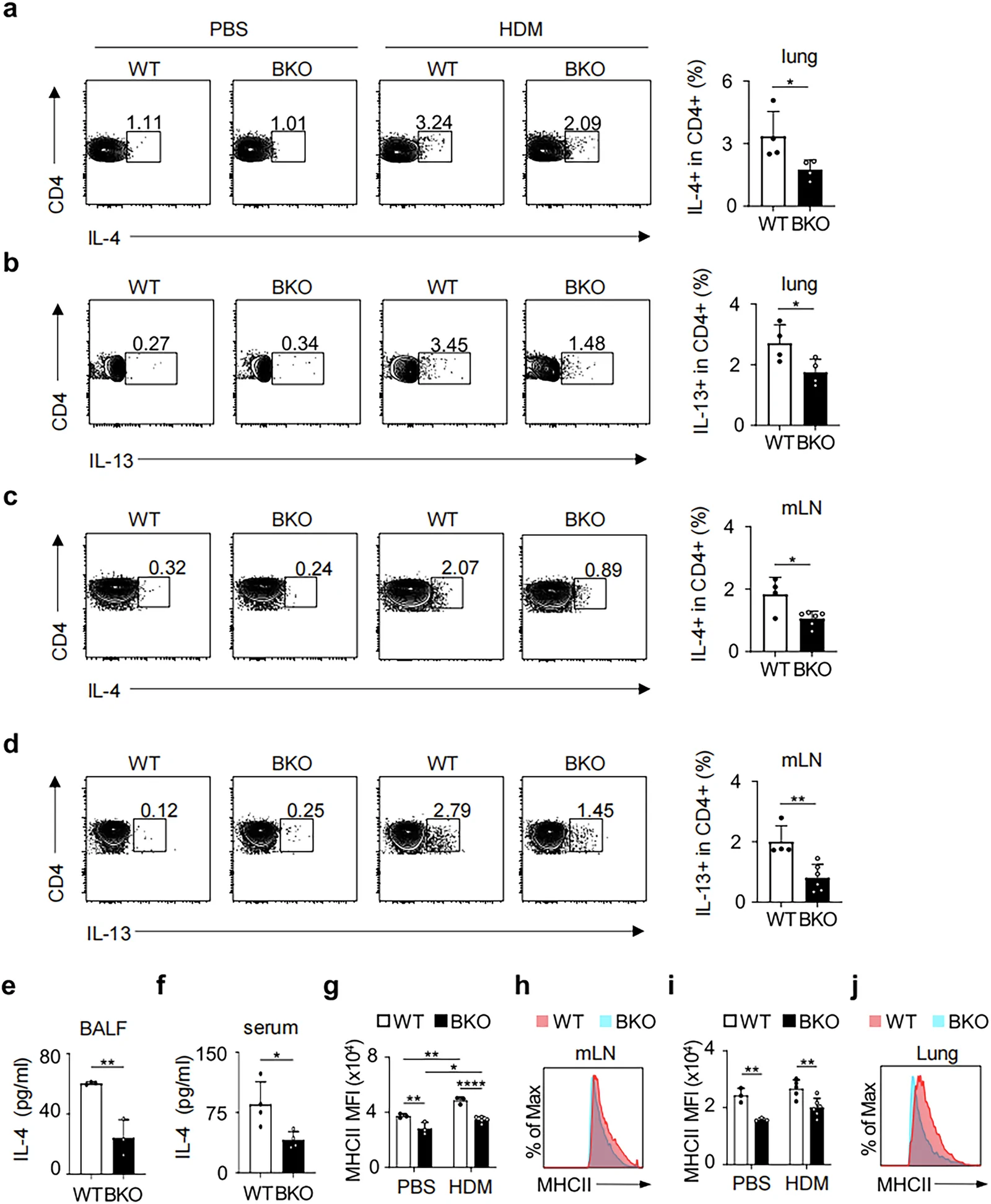
B cell-intrinsic Ssu72 regulates T_H2_ immune response in HDM-induced asthma. **a**, **b** Flow cytometric analysis of lung CD4^+^ T cells producing IL-4 (**a**) and IL-13 (**b**). The percentages of IL-4^+^ (**a**) and IL-13^+^ (**b**) cells among CD4^+^ T cells from lungs in WT and Ssu72-BKO mice in the HDM-induced asthma model (*n* = 4 per group). **cd** Flow cytometric analysis of mLN CD4^+^ T cells producing IL-4 (**c**) and IL-13 (**d**). Percentages of IL-4^+^ cells in **c** and IL-13^+^ cells in **d** among CD4^+^ T cells from the mLN of WT and Ssu72-BKO mice in the HDM-induced asthma model (*n* = 4 per group) are shown. **e**, **f** Total IL-4 concentrations in the BALF (**e**) and serum (**f**) of the HDM-induced asthma model of WT and Ssu72-BKO mice (*n* = 3 or 4 per group). **g** The mean fluorescence intensities (MFIs) of MHC II on mLN B cells from WT and Ssu72-BKO mice in the PBS-injected group and HDM-induced asthma model group (*n* = 3–5 per group). **h** Representative histogram of MHC II on mLN B cells from WT and Ssu72-BKO mice with HDM-induced asthma. **i** MFI of MHC II on lung B cells from WT and Ssu72-BKO mice in the PBS-injected group and HDM-induced asthma model group (*n* = 3–6 per group). **j** Representative histogram of MHC II on lung B cells from WT and Ssu72-BKO mice with HDM-induced asthma. Data are presented as mean ± s.d. *P < 0.05, **P < 0.01, ***P < 0.001, ****P < 0.0001, Student’s *t*-test, two-way ANOVA followed by Bonferroni’s post hoc test.

Given the central role of type 2 cytokines in allergic airway inflammation, we investigated whether the deletion of Ssu72 in B cells also influences cytokine production by CD4^+^ T cells. We evaluated IL-4- or IL-13-producing CD4^+^ T cells upon stimulation of single-cell suspensions from the lungs and mLNs of WT and Ssu72-BKO mice with PMA–ionomycin for 5 h. After asthma induction, the percentages of CD4^+^ T cells producing IL-4 and IL-13 in the lungs (Fig. 7a, b) and mLNs (Fig. 7c, d) were reduced in Ssu72-BKO mice compared to WT mice. Moreover, the concentrations of IL-4 in the BALF and serum were lower in Ssu72-BKO mice than in WT mice with HDM-induced asthma (Fig. 7e, f). We next assessed whether Ssu72 deficiency affects the expression of MHC II and costimulatory molecules on B cells. After HDM induction, the expression levels of MHC II on mLN B cells were upregulated in both WT and Ssu72-BKO mice compared to PBS-injected controls (Fig. 7g). However, regardless of treatment, B cells in the mLNs (Fig. 7g, h) and lungs (Fig. 7i, j) of Ssu72-BKO mice consistently exhibited lower MHC II expression than those from WT mice. In addition, we found the expression levels of CD40 on mLN B cells were lower in Ssu72-BKO mice than WT mice, while CD40 expression on lung B cells remained comparable after HDM induction (Supplementary Fig. 10a). Furthermore, the expression levels of CD80 on B cells showed no significant difference between WT and Ssu72-BKO mice in either the lungs or mLNs following PBS or HDM injection (Supplementary Fig. 10b). By contrast, CD86 expression on both mLN and lung B cells were increased in Ssu72-BKO mice compared to WT controls regardless treatment (Supplementary Fig. 10c), which may be due to the environmental alteration in vivo or a compensatory response to increased mCD23 expression. Taken together, these findings suggest that Ssu72 deficiency in B cells attenuates type 2 lung inflammation and asthma.

## Discussion

B cells contribute to the pathogenesis of allergic asthma by producing IgE^44,45^. However, B cell-intrinsic regulation of their function in allergic asthma remains poorly defined. Here, we demonstrate the critical role played by Ssu72 in B cells during allergic asthma. Ssu72 has been identified as having multiple roles, including acting as a transcription factor, phosphatase and mRNA transcription regulator^46^. Although the immunological functions of Ssu72 have been documented in T cells and macrophages, its role in B cells remains largely unexplored. The present study shows that Ssu72 critically influences B cell function by upregulating Ssu72 expression upon activation, regulating class switching to IgG1 and IgE and shedding CD23 through ADAM10 upregulation. These findings indicate that specific targeting of Ssu72 in B cells is an excellent candidate strategy for treating allergic asthma.

Ssu72 has emerged as an important regulator in immune cells. For example, it was suggested to be a critical regulator of the differentiation of CD4^+^ T cells into peripheral regulatory T cells via Foxp3^29^. In addition, the loss of Ssu72 in CD4^+^ T cells led to an imbalance between regulatory T cells and effector T cells, thus enhancing susceptibility to colitis. In addition, Ssu72 has also been shown to play a critical role in alveolar macrophages during the development of allergic asthma^30^. Mice with macrophage-specific deletion of Ssu72 exhibited defects in the development and maturation of lung-resident alveolar macrophages and were less responsive to the experimental development of asthma^30^. In our study, Ssu72 expression was strongly induced in B cells stimulated with anti-CD40 or IL-4 and in B cells infiltrating HDM-induced asthmatic lungs. Furthermore, B cell-specific Ssu72-knockout mice exhibited markedly attenuated airway inflammation and reduced IgE/IgG1 levels in HDM-induced asthma model. Interestingly, B cell-specific Ssu72-knockout mice showed competent humoral immunity, with preserved GC formation and antigen-specific antibody responses following immunization with NP-KLH, NP-LPS and NP-Ficoll. This suggests that Ssu72 deficiency does not profoundly impair B cell antibody responses. Instead, the reduced IgE/IgG1 responses in HDM-induced asthma may reflect a context-dependent requirement for B cell-expressed Ssu72 in allergic airway inflammation, particularly in the context of mucosal T_H2_ immunity. These findings support the potential role of Ssu72 as a therapeutic target for allergic asthma without broadly compromising systemic humoral immunity.

CSR is essential for generating antibody isotypes other than IgM. B cells preferentially undergo CSR to IgG1 and IgE with the assistance of T_FH_ cells within GCs under T_H2_ conditions, resulting in the production of IgG1 and IgE. Our data suggest that Ssu72 regulates allergic responses by influencing CSR intrinsically and modulating the GC–T_FH_ interaction. Notably, we observed significantly reduced populations of GC B cells and T_FH_ cells in the absence of Ssu72 in B cells, indicating that B cell-intrinsic Ssu72 is crucial for GC responses. Moreover, isolated Ssu72-deficient B cells also displayed reduced switching to IgG1 and IgE and secretory antibody production, further supporting the B cell-intrinsic role of Ssu72 in CSR.

Mechanistically, we observed increased AMPK phosphorylation in Ssu72-deficient B cells. AMPK is a crucial energy sensor that regulates various aspects of intracellular metabolism, prioritizing energy conservation by restraining anabolism^20^. AMPK directly inhibits mTORC1 activity, which is a central integrator in many biosynthetic pathways, especially protein synthesis and cellular growth^20,47^. Although AMPK has been reported to be dispensable for systemic T cell-dependent humoral immune response, AMPK may still play an important role in other immune contexts, such as mucosal immune responses^25^. This context-dependent function of AMPK is consistent with our observation in B cell-specific Ssu72-knockout mice, in which HDM-induced IgG1 and IgE responses were impaired, whereas systemic humoral responses were preserved. In addition, mTORC1 activity is believed to play a crucial role in regulating various B cell functions. Previous studies have demonstrated that the absence of mTORC1 or its inhibition by rapamycin impairs B cell proliferation and CSR, while also reducing both primary and secondary antibody responses^48,49^. In addition, mTORC1 signaling was also required to maintain GC responses to generate high-affinity antibodies^49,50^. Of note, mTORC1 activity was enhanced through the deletion of AMPK in B cells, leading to an increase in antibody synthesis^24^. Interestingly, our study observed reduced activity of mTORC1 signaling in Ssu72-deficient B cells, as evidenced by lower expression levels of p-S6K and p-S6. This reduction may explain the impaired ability to produce IgG1 and IgE. Diminished mTORC1 signaling could restrict the anabolic and translational programs necessary for class-switched B cells to survive and produce antibodies. Furthermore, we observed a physical interaction between Ssu72 and AMPKα. Because Ssu72 functions as a phosphatase, this interaction suggests that Ssu72 may dephosphorylate AMPKα in B cells. Direct phosphatase assays or studies using catalytically inactive Ssu72 mutants will be required to further confirm that AMPKα is a direct substrate of Ssu72 phosphatase.

CD23 is suggested to be an important regulator of IgE production. As a low-affinity IgE receptor, mCD23 plays a negative regulatory role in this process. Notably, CD23-deficient mice have been reported to exhibit increased IgE production^16,17^. However, sCD23 positively regulates IgE production. It has been reported that the anti-CD23 stalk monoclonal antibody 19G5, which disrupts the trimerization of CD23, resulted in a substantial increase of 150-fold in sCD23 and reduced B cell mCD23 expression by more than 90%. This change led to a two- to threefold increase in IgE levels in mice^18^. In our study, Ssu72 deficiency in B cells resulted in decreased generation of sCD23, while mCD23 expression on B cells increased during HDM-induced asthma. The observed reduction in IgE levels during this condition was probably due to the diminished production of sCD23. ADAM10 is a protease that cleaves mCD23 to generate sCD23 and represents a potential therapeutic target for asthma. The administration of an ADAM10 inhibitor reduced IgE production and T_H2_ immune responses in a murine asthma model^51^. Moreover, a deficiency of ADAM10 in B cells profoundly suppressed CD23 cleavage^52^. As Ssu72-deficient B cells exhibited reduced ADAM10 induction in HDM-induced asthma, the diminished generation of sCD23 in these cells can be attributed to the failure to induce ADAM10 in activated Ssu72-deficient B cells. Moreover, MMP9 has been implicated in the LPS-induced release of sCD23 from B cells, as LPS increased MMP9 expression but failed to induce CD23 cleavage in MMP9^−/−^ cells. In our study, the deletion of Ssu72 in B cells resulted in reduced MMP9 expression levels both in HDM-induced asthma and under LPS stimulation, indicating that Ssu72 is involved in the regulation of MMP9. There appears to be a positive correlation between Ssu72 and MMP9. However, despite compelling evidence that Ssu72 regulates CD23 shedding through ADAM10 and MMP9, the mechanisms by which Ssu72 influences the expression of ADAM10 and MMP9 remain unclear. Therefore, further investigation is needed to enhance our understanding of the molecular mechanisms involved in Ssu72-mediated regulation of ADAM10 and MMP9. In addition, Ssu72 is involved in both AMPK–mTORC1 axis and CD23-shedding axis; however, the relationship between these two pathways remains to be determined.

Allergic asthma develops through sensitization and challenge phases. The challenge phase, also known as the effector phase, triggers clinical manifestations upon re-exposure to the same allergen^53^. Recent articles have suggested that B cells play a critical role in both phases. The adoptive transfer of various B cell subsets or modified B cells into B cell-deficient mice before the challenge phase can either promote or suppress the asthmatic response^54–56^, indicating that targeting B cells offers a potential strategy for developing novel therapies. In this study, our findings suggest that B cell-expressed Ssu72 may be involved in both the sensitization and challenge phases, as evidenced by a reduced number of IgE class-switched B cells in Ssu72-BKO mice and diminished effector functions of Ssu72-deficient B cells in HDM-induced asthma, respectively.

Type 2 cytokines play a critical role in the development and exacerbation of asthma, highlighting their crucial involvement in the pathogenesis of asthma^4,57^. Cytokines, including IL-4 and IL-13, promote B cells to undergo CSR to IgE and IgG1. In the present study, we observed fewer increases in IL-4/IL-13-producing CD4^+^ T cells in mLNs and lungs, as well as reduced IL-4 levels in BALF and serum in asthma-induced Ssu72-BKO mice compared to asthma-induced WT mice. Reduced type 2 cytokine production in the lungs may contribute to the alleviation of local allergic inflammation, while reduced IL-4 production in mLNs may limit B cell CSR to IgE. The absence of Ssu72 in B cells not only leads to intrinsic defects of B cell function but also affects the interacting cells including CD4^+^ T cells, which may further inhibit the production of pathogenic B cells. By contrast, B cells have also been shown to have the ability to present antigen to T cells, thus sustaining T_H2_-mediated inflammation in asthma^44^. It was previously shown that the deletion of B cells before the challenge phase reduced T_H2_-related cytokine levels in HDM-induced asthma. In our study, we observed reduced upregulation of MHC II in Ssu72-deficient B cells upon activation alongside abnormally increased levels of CD86 expression. While CD86 provides an important costimulatory signal that enhances T cell activation, the antigen-presenting capacity mediated by MHC II is crucial for effective antigen-specific CD4^+^ T cell activation. Therefore, reduced MHC II expression on B cells may limit the antigen-specific T cell response, despite elevated CD86 levels. The abnormal upregulation of CD86 may partly result from the aggregation of CD23 on the cell membrane, as previous studies have suggested a potential link between CD23 and CD86 expression^58^. However, there is a lack of direct evidence regarding whether the reduced T_H2_ response ability is caused by the deletion of Ssu72 in B cells. Further investigations are required to elucidate the role of Ssu72 expressed in B cells in T–B cell cooperative interactions.

**Fig. 8:**
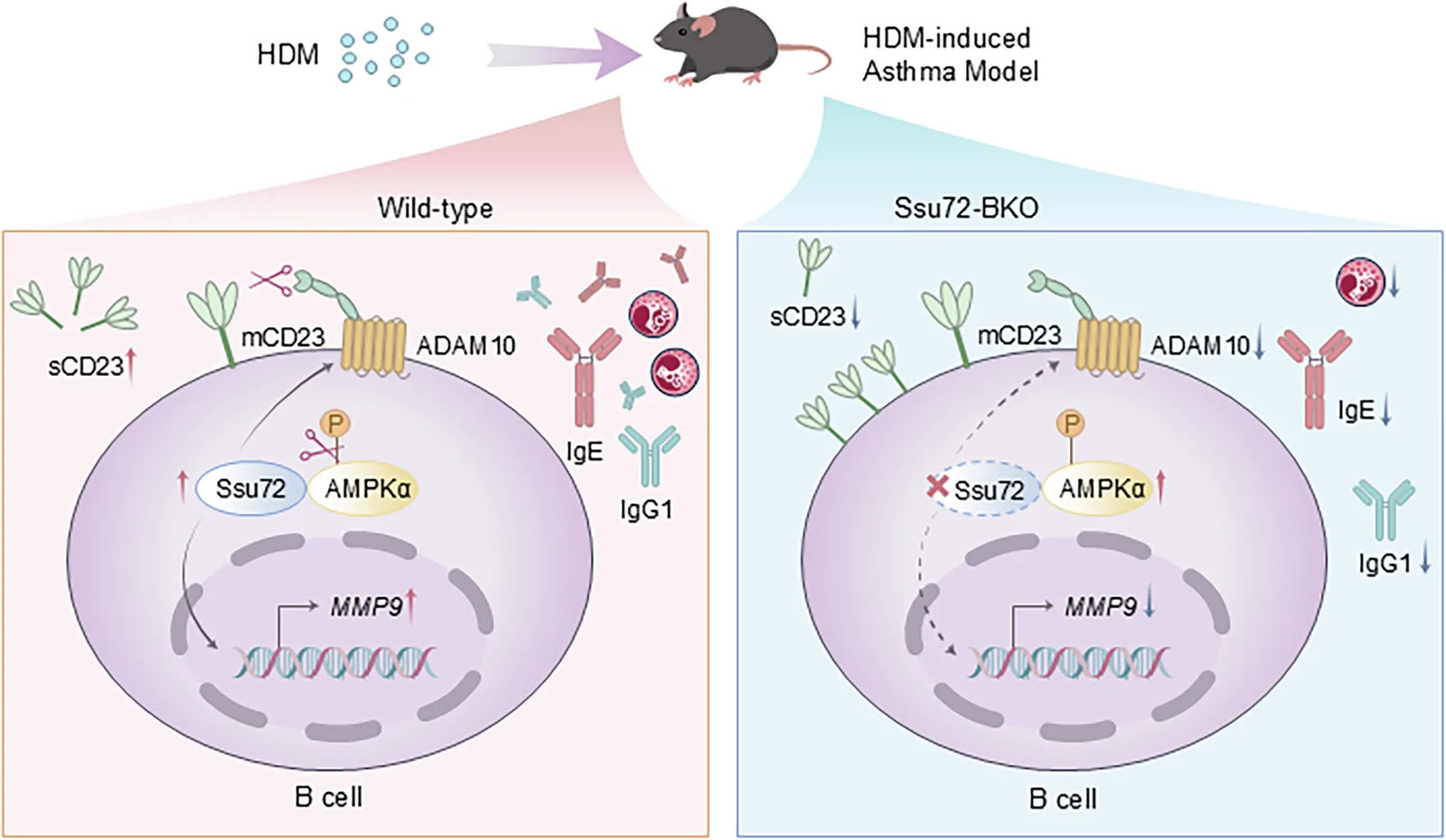
A schematic overview of the intrinsic mechanism underlying the role of B cell-intrinsic Ssu72 in the HDM-induced asthma model. B cell-expressed Ssu72 is upregulated in the HDM-induced asthma. Ssu72 regulates B cell class switching to IgG1 and IgE, as well as CD23 shedding through the induction of ADAM10 and MMP9. Mechanistically, Ssu72 may mediate AMPK–mTORC1 signaling pathway through dephosphorylation of AMPKα.

We collectively demonstrate the role of Ssu72 in B cells during the pathogenesis of allergic asthma. Our findings reveal that B cell-expressed Ssu72 is crucial for several B cell functions, including CSR to IgG1 and IgE and CD23 shedding, as shown in the HDM-induced asthma model. Our results also demonstrate that Ssu72 may dephosphorylate AMPKα to regulate the AMPK–mTORC1 signaling pathway. This study highlights Ssu72 as a novel regulator of B cell function and emphasizes the potential of targeting Ssu72 in B cells for the treatment of allergic asthma (Fig. 8).

## Supplementary information

Supplementary Information

## Data Availability

The data generated in this study, along with any additional information or reagents, are available from the corresponding author upon reasonable request.
