# Supplementary material for: B cell-intrinsic Ssu72 phosphatase is a key regulator of allergic asthma
Source: Exp Mol Med. 2026 Oct 5;58(10):3457–71. doi: 10.1038/s12276-026-01859-0 (PMC13737138; doi:10.1038/s12276-026-01859-0)
Supplement: Supplementary file 1 [file 12276_2026_1859_MOESM1_ESM.pdf]

Supplementary Information

B cell-intrinsic Ssu72 phosphatase is a key regulator of allergic asthma

Seo-Young Koo[1*], Naiyu Wang[1*], Min-Hee Kim[1], Sujin Lee[2,3], Tae Jin Kim[2,3], Chang-Woo Lee[1,4]

[1]Department of Molecular Cell Biology, Samsung Medical Center, Sungkyunkwan University School of Medicine, Suwon 16419, Republic of Korea; [2]Department of Immunology, Sungkyunkwan University School of Medicine, Suwon 16419, Republic of Korea; [3]Personalized Cancer Immunotherapy Research Center, Sungkyunkwan University, Suwon 16419, Republic of Korea; [4]SKKU Institute for Convergence, Sungkyunkwan University, Suwon 16419, Republic of Korea

Corresponding authors: Chang-Woo Lee, PhD, Department of Molecular Cell Biology, Samsung Medical Center, Sungkyunkwan University School of Medicine, 2066 Seobu-ro, Jangan-gu, Suwon-si, Gyeonggi-do, Republic of Korea. Phone: +82-31-299-6153; Email: cwlee1234@skku.edu; Or: Tae Jin Kim, PhD, Department of Immunology, Sungkyunkwan University School of Medicine, Suwon 16419, Republic of Korea. Phone: +82-31-299-6161; Email: tjkim@skku.edu

*SYK and NW equally contributed to this study as first authors.

This file includes:

Supplementary Table 1

Supplementary Figures 1 to 10

Supplementary Table 1. List of antibodies used in this study

Antibodies	Catalog #	Clone	Application	Supplier
Ssu72	12816	D3I2D	Western Blot, Immuno-fluorescence staining	Cell Signaling
ADAM10	14194		Western Blot	Cell Signaling
Phospho-AMPK alpha (Thr172)	50081	D4D6D	Western Blot	Cell Signaling
AMPK alpha	2532		Western Blot, Immuno-precipitation	Cell Signaling
S6 Ribosomal Protein	2217	5G10	Western Blot	Cell Signaling
Phospho-S6 Ribosomal Protein (Ser 235/236)	4858	D57.2.2E	Western Blot	Cell Signaling
Phospho-p70 S6 Kinase (Thr389)	9234	108D2	Western Blot	Cell Signaling
p70 S6 Kinase Antibody	9202		Western Blot	Cell Signaling
Myc-Tag	2276	9B11	Western Blot, Immuno-precipitation	Cell Signaling
HA-Tag	3724	C29F4	Western Blot	Cell Signaling
β-actin	4967		Western Blot	Cell Signaling
Normal Rabbit IgG	2729		Immunoprecipitation	Cell Signaling
Annexin V Apoptosis Detection Kits	88-8007-72		Flowcytometry	eBioscience
Live/dead-APC cy7	L34976		Flowcytometry	Invitrogen
CD16/CD32	14-0161-82	93	Flowcytometry	eBioscience
CD45-PE	12-0451-82	30-F11	Flowcytometry	eBioscience
CD45- PerCP Cy5.5	45-0451-82	30-F11	Flowcytometry	eBioscience
CD45-BV421	404-0451-82	30-F11	Flowcytometry	eBioscience
CD3-PE Cy7	25-0038-42	UCHT1	Flowcytometry	eBioscience
CD4-FITC	11-0041-82	GK1.5	Flowcytometry	eBioscience
CD45R (B220) -FITC	11-0452-82	RA3-6B2	Flowcytometry	eBioscience

CD45R (B220) -PE	12-0452-82	RA3-6B2	Flowcytometry	eBioscience
CD45R (B220) - PerCP Cy5.5	45-0452-82	RA3-6B2	Flowcytometry	eBioscience
CD45R (B220) -PE cy7	25-0460-82	HIS24	Flowcytometry	eBioscience
CD45R (B220) -APC	47-0452-82	RA3-6B2	Flowcytometry	eBioscience
CD45R (B220) -APC Cy7	47-0452-82	RA3-6B2	Flowcytometry	eBioscience
CD69-PE	12-0691-82	H1.2F3	Flowcytometry	eBioscience
CD86-PE Cy7	25-0862-82	GL1	Flowcytometry	eBioscience
CD86-APC	17-0869-42	B7-2	Flowcytometry	eBioscience
MHCII-FITC	14-5321-82	M5/114.15.2	Flowcytometry	eBioscience
CD40-PE	12-0401-82	1C10	Flowcytometry	eBioscience
CD23-PE	12-0238-42	EBVCS2	Flowcytometry	eBioscience
CD185(CXCR5)-PE	12-9185-42	MU5UBEE	Flowcytometry	eBioscience
CD279 (PD-1)-PE Cy7	25-2799-42	eBioJ105 (J105)	Flowcytometry	eBioscience
CD11c-PE Cy7	25-0114-82	N418	Flowcytometry	eBioscience
Ly-6G/Ly-6C Gr1-APC	14-5931-82	RB6-8C5	Flowcytometry	eBioscience
CD11b-APC Cy7	17-0112-82	M1/70	Flowcytometry	eBioscience
CD95 (APO-1/Fas)-PE	12-0951-81	15A7	Flowcytometry	eBioscience
GL7-APC	14-5902-82	GL-7 (GL7)	Flowcytometry	eBioscience
CD138-APC	17-1389-42	DL-101	Flowcytometry	eBioscience
Ki67-FITC	11-5698-82	SolA15	Flowcytometry	eBioscience
IL-4-APC	17-7041-81	11B11	Flowcytometry	eBioscience
IL-13-PE	12-7133-81	eBio13A	Flowcytometry	eBioscience
IgE-FITC	11-5992-81	23G3	Flowcytometry	eBioscience

IgG1-PE	406607	RMG1-1	Flowcytometry	eBioscience
CD45R/B220	553084	RA3-6B2	Immunofluorescence staining, Immunohistochemistry	BD Bioscience
Eosinophil cationic protein (ECP)	orb156688		Immunohistochemistry	Biorbyt
Goat anti-Rat IgG Alexa Fluor® 488	A-11006		Immunofluorescence staining	Invitrogen
Goat anti-rabbit IgG Alexa Fluor® 568	A-11011		Immunofluorescence staining	Invitrogen

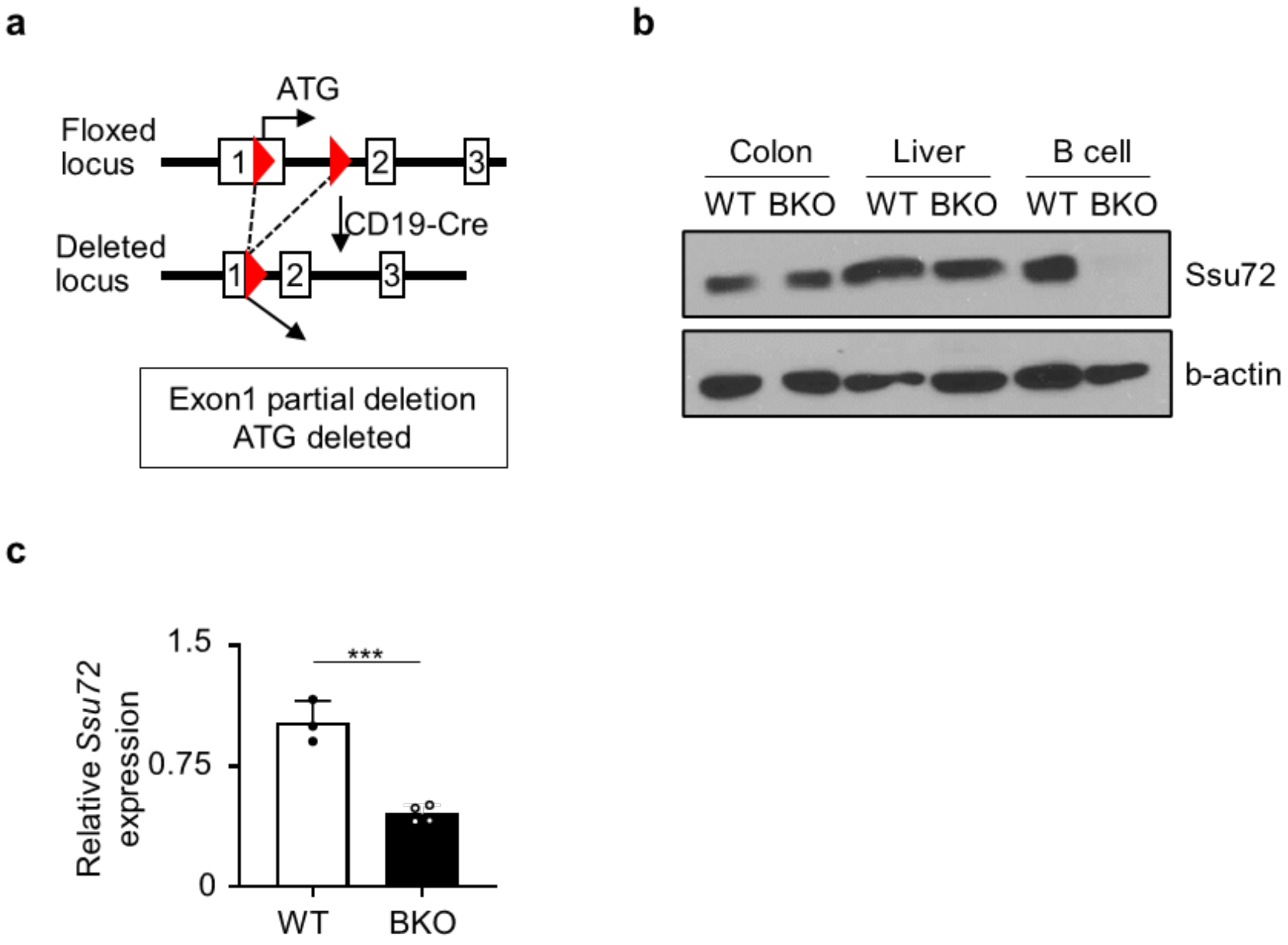

Supplementary Fig. 1 Generation of B cell-specific Ssu72 KO mice

a Schematic diagram of the strategy used to generate B cell-specific Ssu72 KO mice. The Ssu72 floxed allele contains two floxP sites (indicated by red triangles) that flank exon 1, which includes the ATG codon. To generate mice with a B cell-specific deletion of the Ssu72 allele (CD19-Cre; Ssu72$^{f/f}$, Ssu72-BKO mice), mice possessing floxed Ssu72 alleles (Ssu72$^{f/f}$, WT) were crossed with CD19-Cre mice. **b** Ssu72 protein expression was analyzed in colon, liver, and B cells by immunoblotting. **c** The mRNA expression levels of *Ssu72* in WT and Ssu72-BKO sorted B220^{+} B cells were measured and normalized to that of *GAPDH* (n = 3-4 per group). Data are presented as mean ± SD. *$p < 0.05$ **$p < 0.01$ ***$p < 0.001$, Student's *t* test.

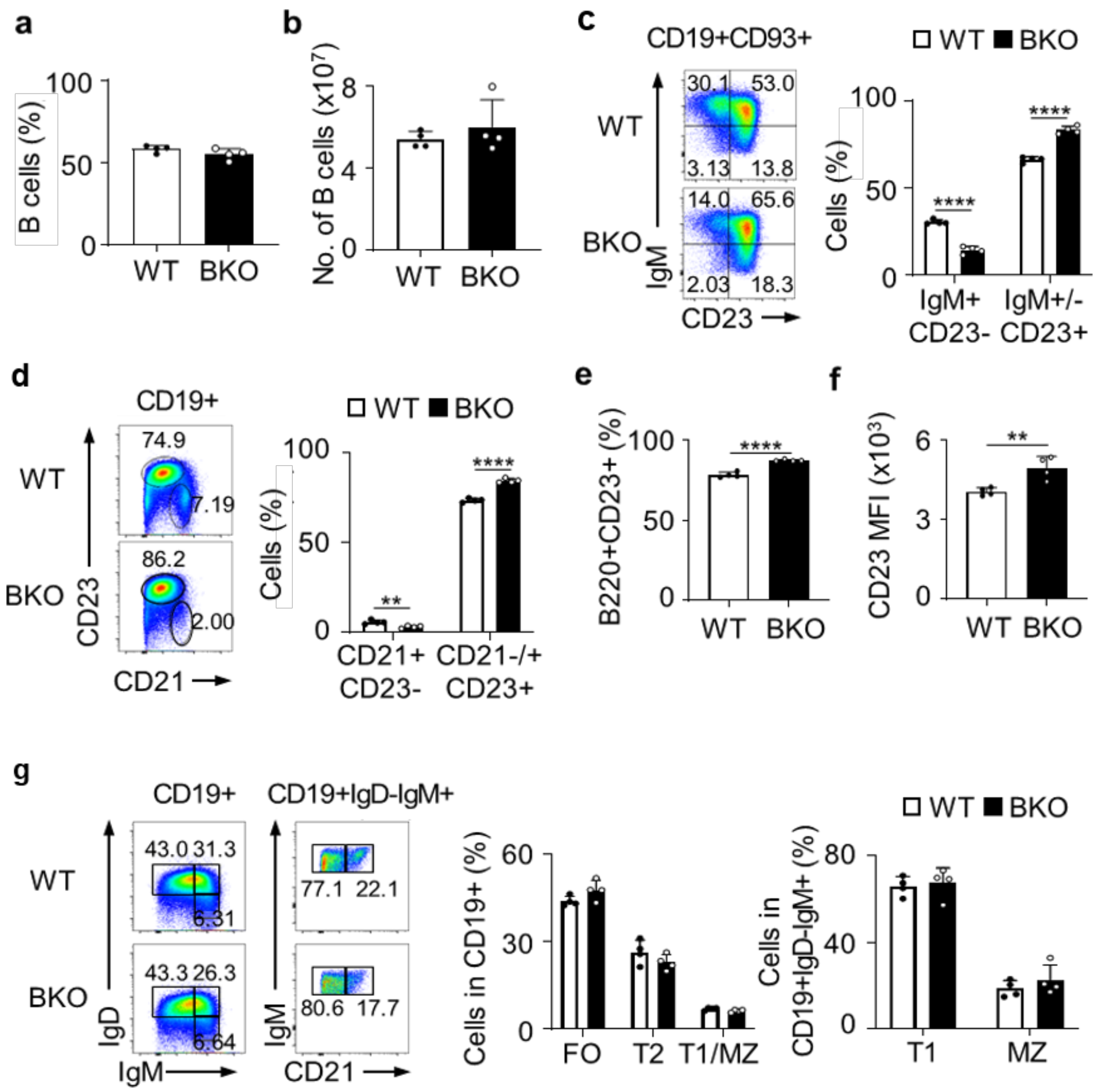

Supplementary Fig. 2 Normal B cell development in B cell-specific Ssu72 KO mice

a, b The percentages **(a)** and numbers **(b)** of B220^{+} B cells obtained from the spleens of 8-week-old WT and Ssu72-BKO mice. **c, d** Flow cytometric analysis to determine the subtypes of B cells in spleen of WT and BKO mice. Frequencies of IgM^{+}CD23^{-} cells and IgM$^{+/-}$CD23^{+} cells among CD19^{+}CD93^{+} splenocytes **(c)** and frequencies of CD21^{+}CD23^{-} cells and CD21^{int}CD23^{+} cells among CD19^{+} splenocytes **(d)**. **e** Graph summarizing the frequency of B220^{+}CD23^{+} cells derived from the spleen of WT and BKO mice. **f** Surface expression of CD23 on splenic B220^{+} B cells, represented as mean fluorescence intensity (MFI). **g** Flow

cytometric analysis of subtypes of B cell in spleens from WT and BKO mice. After gating on $CD19^+$ B cells, IgM and IgD were used to define FO B cells ($IgD^{hi}IgM^{lo}$), T2 B cells ($IgM^{hi}IgD^{hi}$). The $IgM^{hi}IgD^{lo}$ population was further separated into $CD21^-$ (T1) and $CD21^+$ (MZ B). Summarized graphs of the percentage of FO, T2, and T1/MZ B cells ($IgM^{hi}IgD^{lo}$) among $CD19^+$ cells (left panels) and T1 ($CD21^-$) and MZ B cells ($CD21^+$) among $CD19^+IgM^{hi}IgD^{lo}$ cells (right panels). (n = 4 per group). Data are presented as mean ± SD. $*p < 0.05$ $**p < 0.01$ $***p < 0.001$, Student's *t* test.

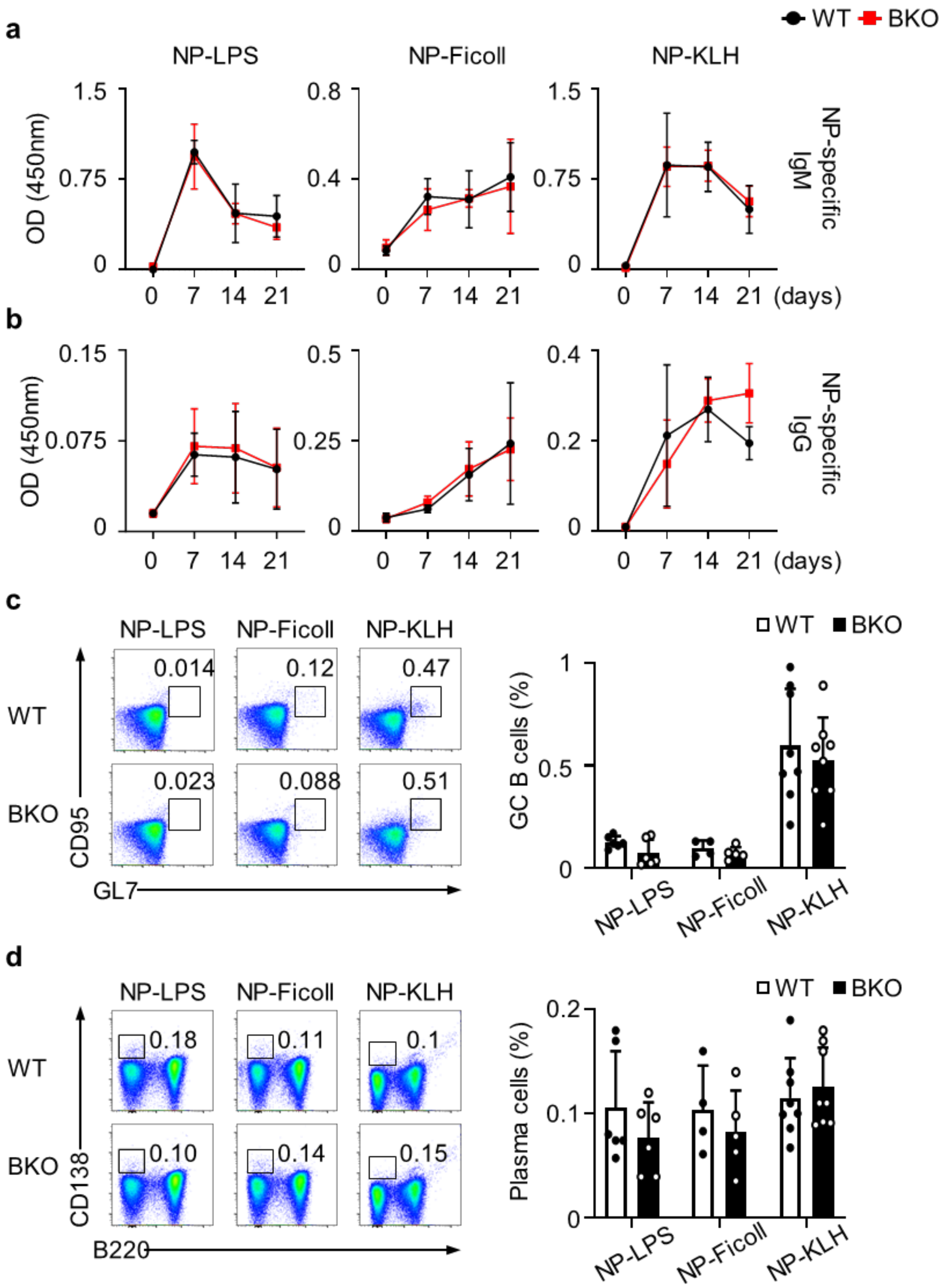

Supplementary Fig. 3 Normal antibody secretion and B cell differentiation after T cell-independent and T cell-dependent antigen exposure in B cell-specific Ssu72 KO mice

a, b The concentrations of NP-specific IgM and IgG in the serum of WT and Ssu72-BKO

mice were measured by ELISA. The mice were immunized intraperitoneally with NP-LPS (100 μg/mL), NP- Ficoll (100 μg/mL), or NP-KLH (100 μg/mL, 1:1 mixed with Alum), and the serum samples were collected on day 0, 7, 14 and 21 days after challenge (n = 3-6 per group). **c, d** Flow cytometric analysis and summarized graphs of the percentages of $CD95^+GL7^+$ GC B cells **(c)** and $CD138^+B220^-$ plasma B cells **(d)** in WT and Ssu72-BKO immunized mice (n = 3-6 per group). Data are presented as mean ± SD. *$p < 0.05$ **$p < 0.01$ ***$p < 0.001$, Student's *t* test.

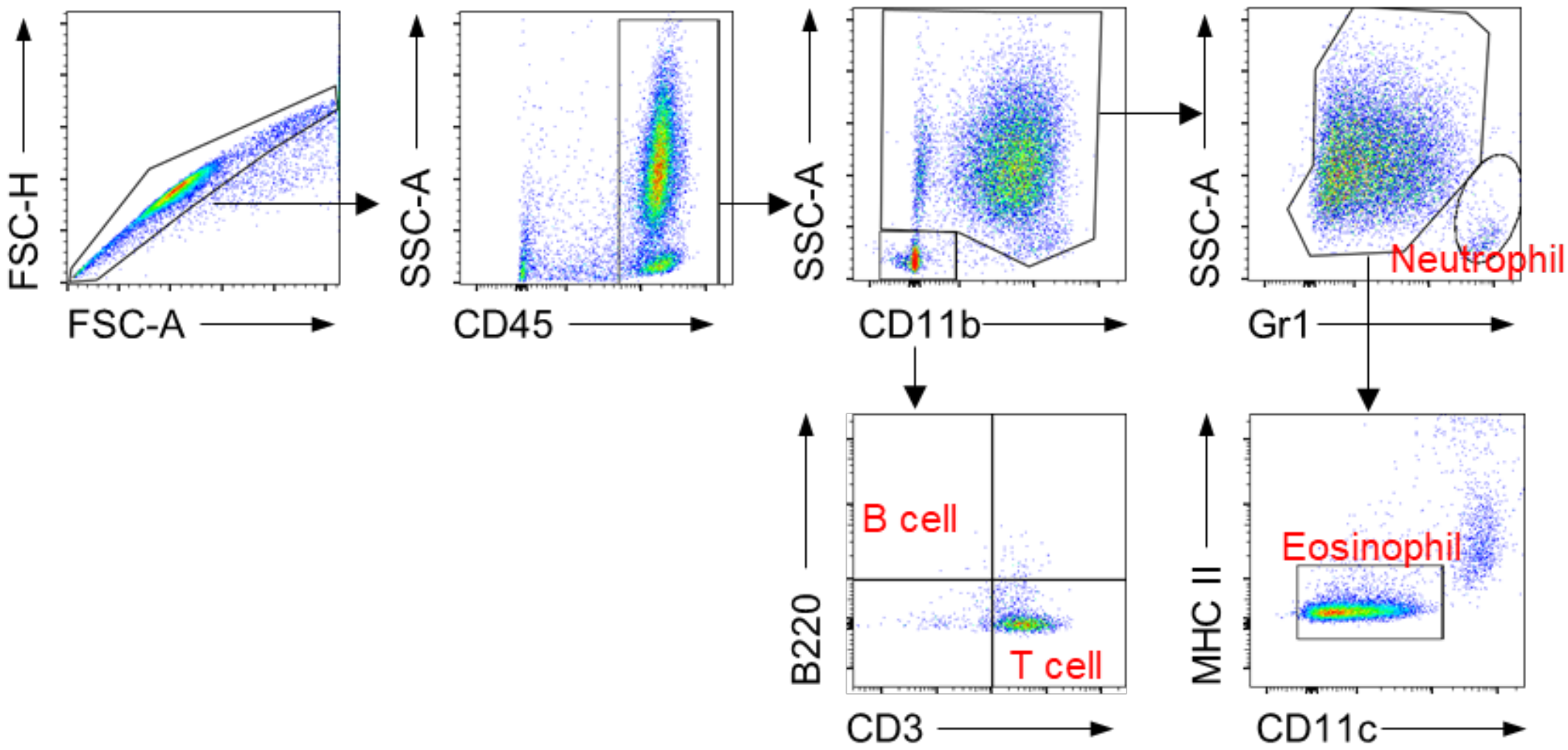

Supplementary Fig. 4 Flow cytometry gating strategies for BALF cells

Gating strategy for neutrophils, eosinophils, T cells, B cells in the BALF. First, CD45$^+$ leukocytes were selected; then lymphocytes were gated on an SSC-A/CD11b plot to discriminate B220$^+$ B cells and CD3$^+$ T cells. Other cells were analyzed with an SSC-A/Gr1 plot to identify neutrophils, and an MHC-II/CD11c plot was used to separate eosinophils from the remaining cells.

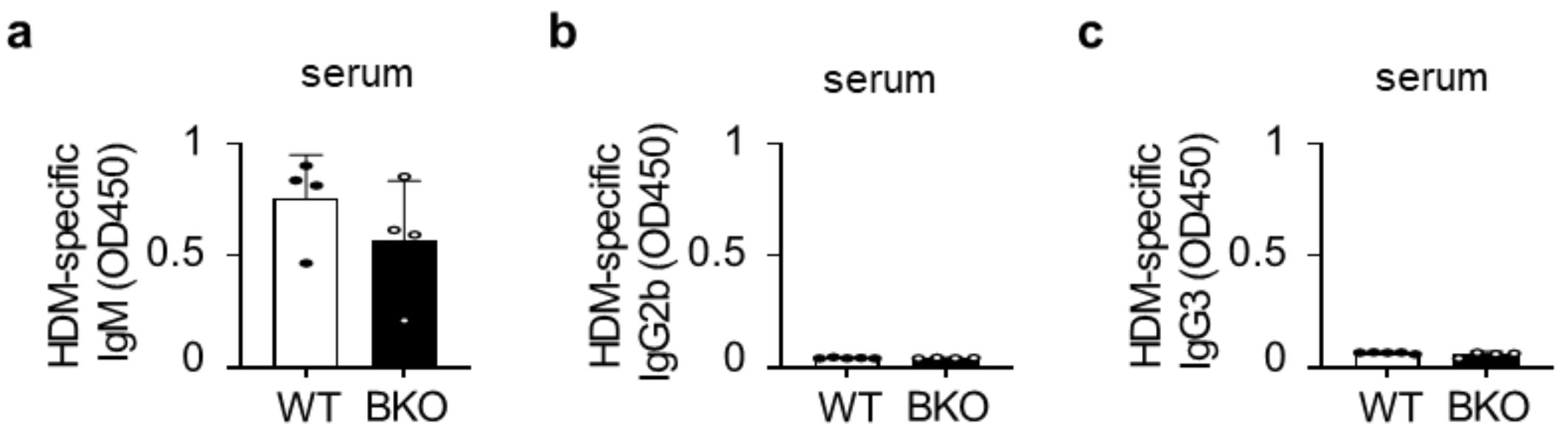

Supplementary Fig. 5 Serum HDM-specific immunoglobulin levels after HDM induced

a, b, c HDM-specific IgM, IgG2b and IgG3 were measured by ELISA (n = 4-5 per group). Data are presented as mean ± SD. *$p < 0.05$ **$p < 0.01$ ***$p < 0.001$, Student's *t* test.

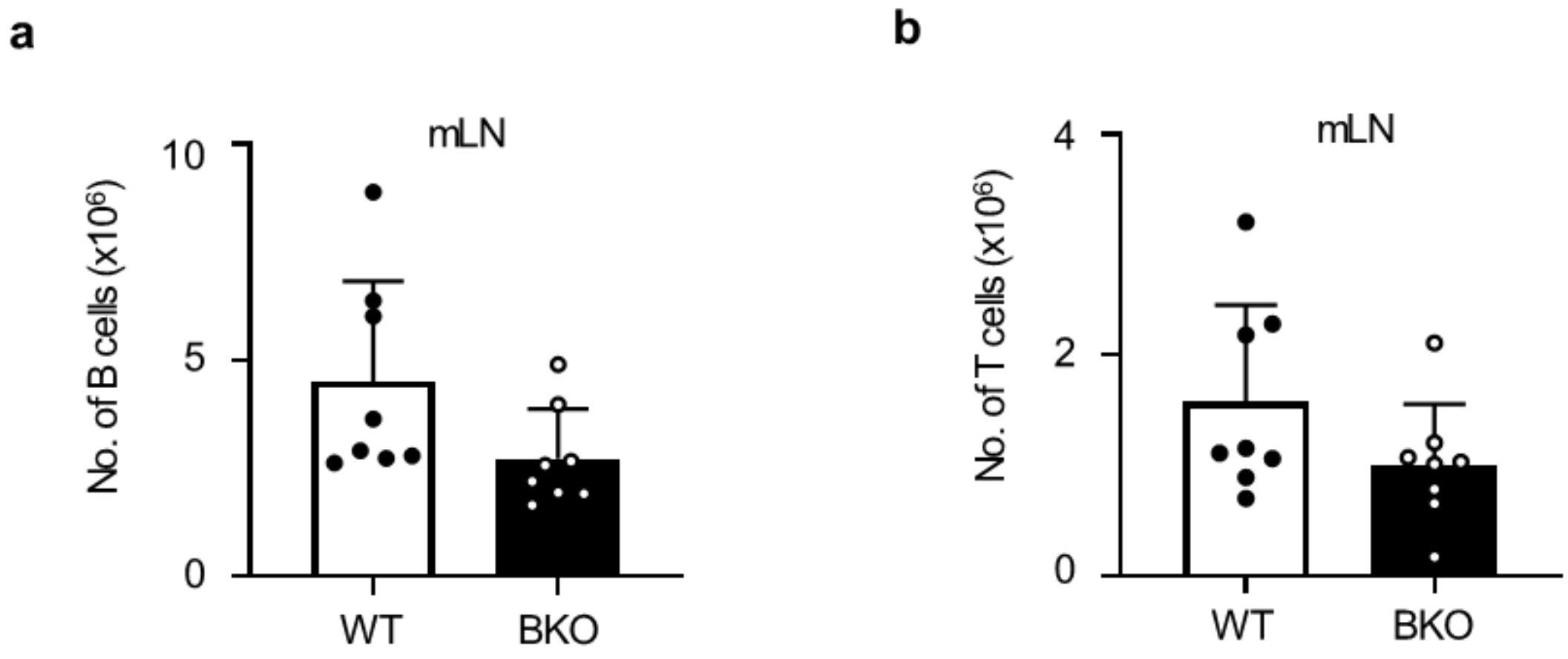

Supplementary Fig. 6 Numbers of B and T cells in mLNs after HDM-induced asthma

a Absolute numbers of B cells in mLNs. **b** Absolute numbers of T cells in mLNs (n = 8 per group). Data are presented as mean ± SD. **p* < 0.05 ***p* < 0.01 ****p* < 0.001, Student's *t* test.

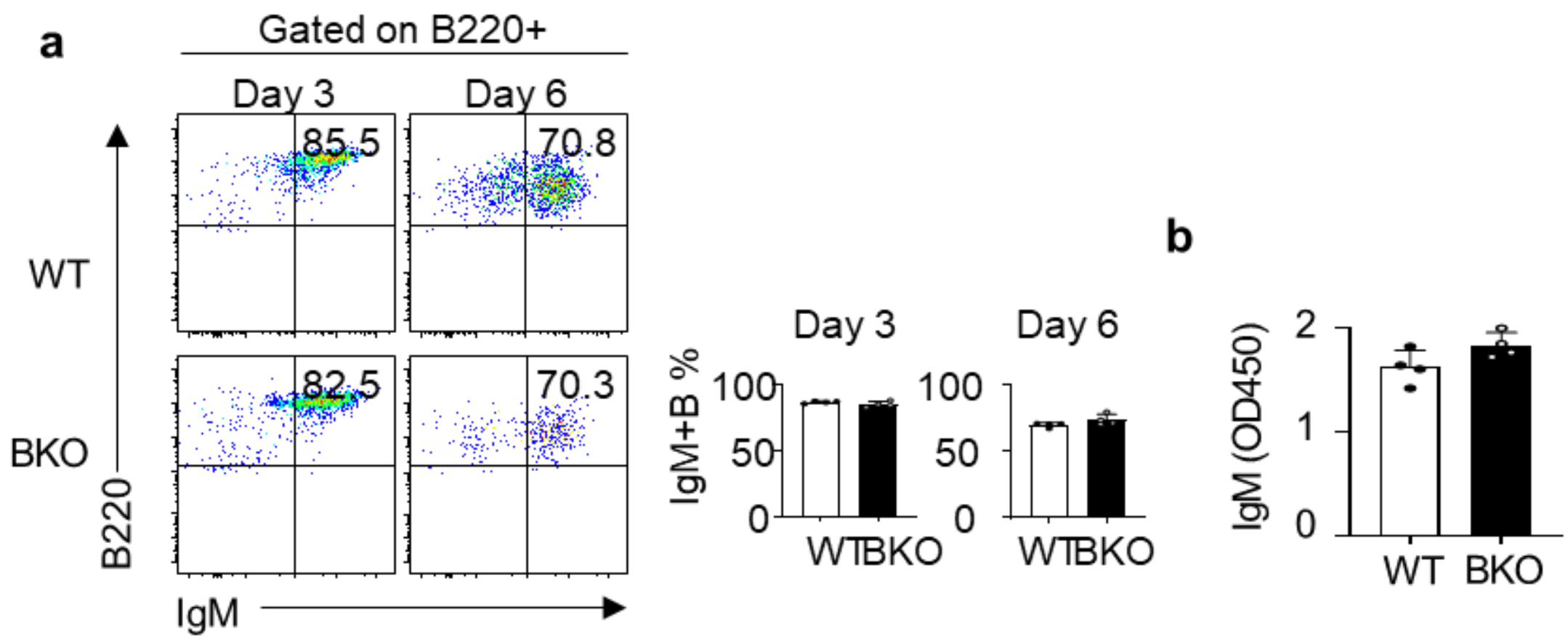

Supplementary Fig. 7 Normal IgM production despite Ssu72 deletion in B cells

a Representative flow cytometry plots of IgM$^+$ B cells from WT and Ssu72-deficient B cells stimulated with LPS (10 μg/mL) plus IL-4 (40 ng/mL) for 3 and 6 days. Percentages of IgM$^+$ B cells in B cells at the indicated time point (n = 4 per group). **b** Levels of IgM in supernatant after stimulation for 6 days (n = 4 per group). Data are presented as mean ± SD. $*p < 0.05$ $**p < 0.01$ $***p < 0.001$, Student's *t* test.

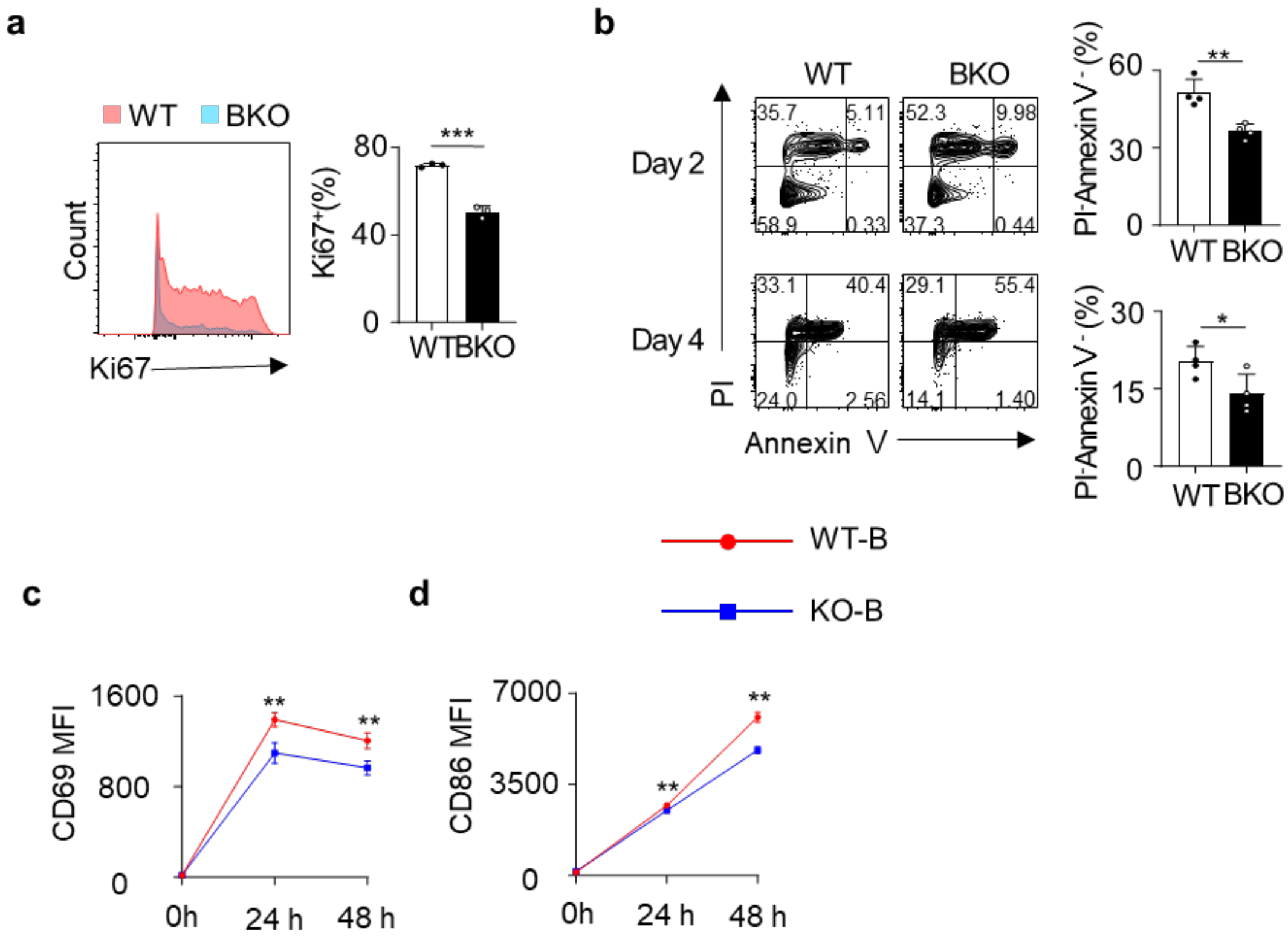

Supplementary Fig. 8 B cell Ssu72 regulates proliferative capacities and survival *in vitro*

a Representative histogram of Ki67 expression on B cells from WT and Ssu72-deficient B cells after stimulation for 4 days. Percentages of $Ki67^+$ B cells among total B cells (n = 3 per group). **b** Representative flow cytometry plots of Annexin V and PI stained cells after stimulation for 2 or 4 days. Percentages of live cells (Annexin V^-PI^-) after stimulation for 2 or 4 days (n = 4 per group). Data are presented as mean ± SD. **c, d** Graph showing MFIs of CD69 (c) and CD86 (d) expression levels in stimulated WT (red) and Ssu72-deficient (blue) B cells at each time point (n = 3-4 per group). Data are presented as mean ± SD. Data are presented as mean ± SD. *$p < 0.05$; **p < 0.01; ***p < 0.001, Student's *t* test.

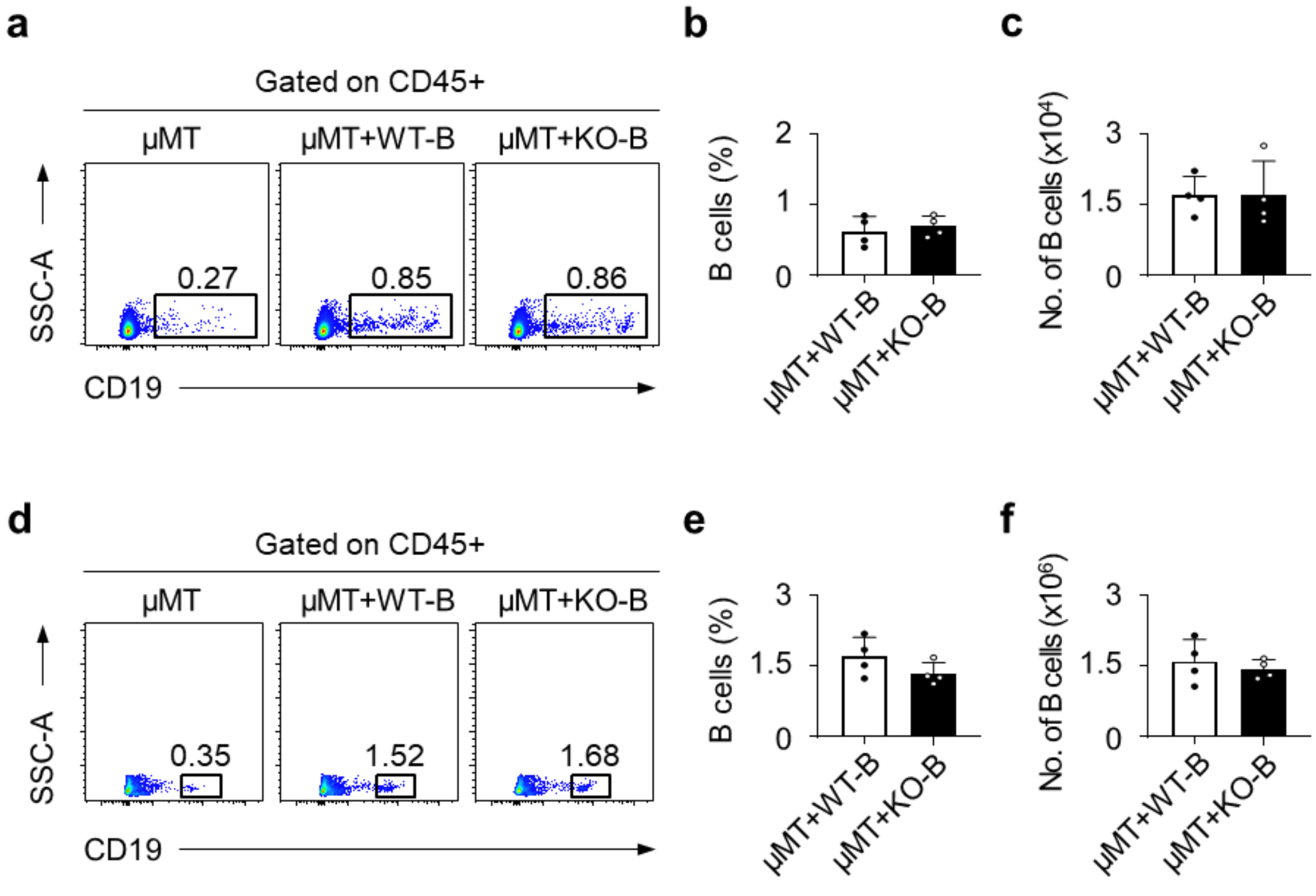

Supplementary Fig. 9 WT B cells and Ssu72-BKO B cells were successfully engrafted into μMT mice after adoptive transfer

a Representative flow cytometry plots of $CD19^+$ B cells in the mLNs of μMT mice receiving WT B cells or Ssu72-deficient B cells. **b, c** The percentages **(b)** and absolute numbers **(c)** of $CD19^+$ B cells in the mLNs of recipient μMT mice (n = 4 per group). **d** Representative flow cytometry plots of $CD19^+$ B cells in the spleens of μMT mice receiving WT B cells or Ssu72-deficient B cells. **e, f** The percentages **(e)** and absolute numbers **(f)** of $CD19^+$ B cells in the spleens of recipient μMT mice (n = 4 per group). Data are presented as mean ± SD. **p* < 0.05 ***p* < 0.01 ****p* < 0.001, Student's *t* test.

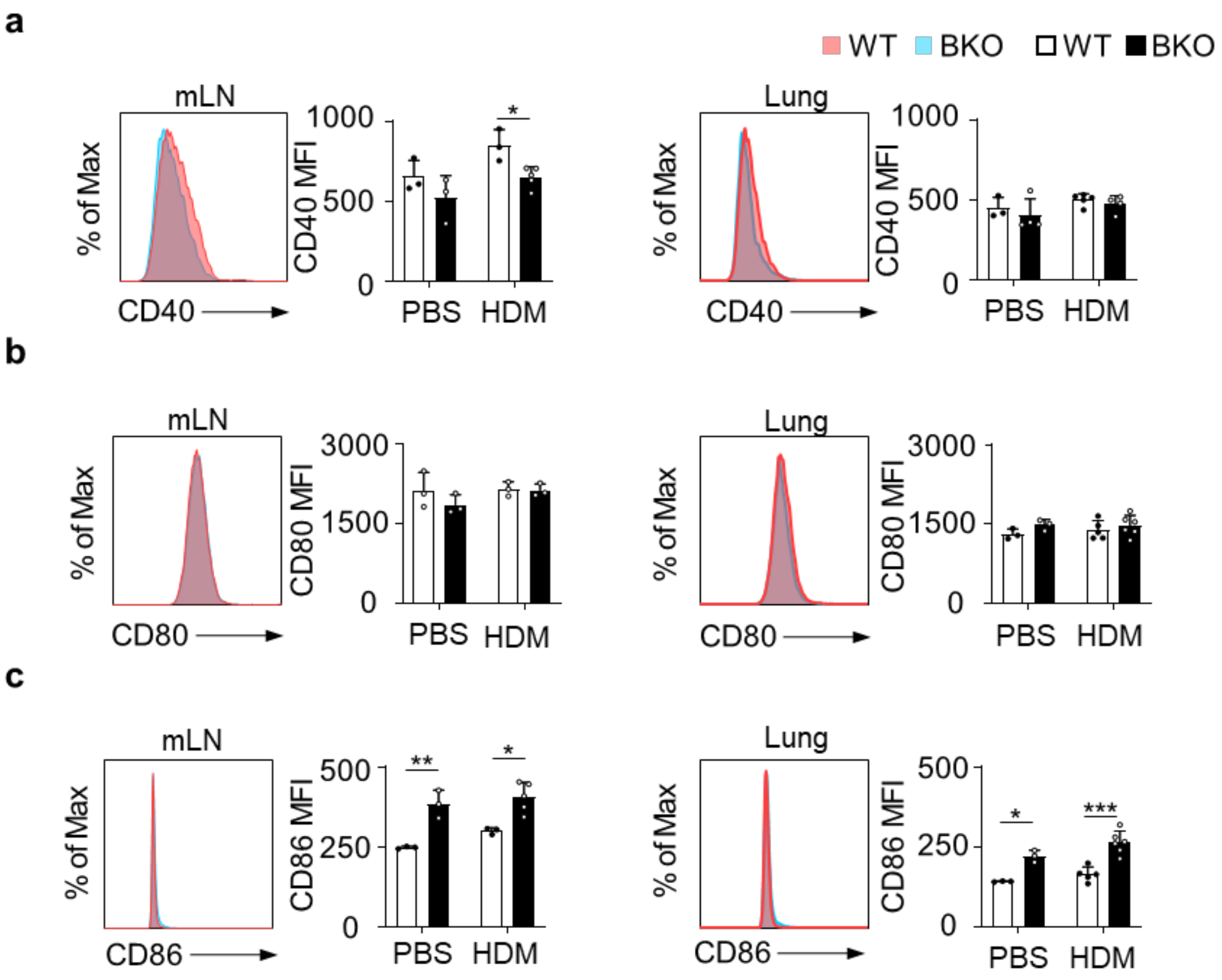

Supplementary Fig. 10 Loss of Ssu72 affects the expression of costimulatory molecules on B cells

a Representative histogram of CD40 on mLNs and lung B cells from WT and Ssu72-BKO mice with HDM-induced asthma. MFI of CD40 on mLNs and lung B cells from WT and Ssu72-BKO mice in the PBS-injected group and HDM-induced asthma model group. **b** Representative histogram of CD80 on mLNs and lung B cells from WT and Ssu72-BKO mice with HDM-induced asthma. MFI of CD80 on mLNs and lung B cells from WT and Ssu72-BKO mice in the PBS-injected group and HDM-induced asthma model group. **c** Representative histogram of CD86 on mLNs and lung B cells from WT and Ssu72-BKO mice with HDM-induced asthma. MFI of CD86 on mLNs and lung B cells from WT and Ssu72-BKO mice in the PBS-injected group and HDM-induced asthma model group (n = 3-6 per

group). Data are presented as mean ± SD. $*p < 0.05$ $**p < 0.01$ $***p < 0.001$, two-way ANOVA followed by Bonferroni's post hoc test.
